# Oil crop supply chains drive rising global biodiversity loss and outsource impacts to the tropics

**DOI:** 10.1038/s43016-026-01375-4

**Published:** 2026-06-19

**Authors:** Shuntian Wang, Livia Cabernard, Martin Bruckner, Maulana Permana Ajie, Stephan Pfister

**Affiliations:** 1 Ecological Systems Design, Institute of Environmental Engineering, ETH Zurich, Zurich, Switzerland; 2 Albert Einstein School of Public Policy, ETH Zurich, Zurich, Switzerland; 3 Sustainability Assessment of Food and Agricultural Systems, School of Management and School of Life Sciences, Technical University of Munich, Munich, Germany; 4 Institute for Ecological Economics, WU Vienna University of Economics and Business, Vienna, Austria; 5 School of Life Sciences, Technical University of Munich, Munich, Germany

**Keywords:** Environmental impact, Sustainability

## Abstract

Oil crop expansion threatens global biodiversity, yet its supply chain impacts remain poorly quantified. Here we analyse biodiversity impacts along oil crop supply chains from 1995 to 2020 using spatially explicit assessments, a hybridized multi-regional input–output framework, enhanced supply chain mapping and structural decomposition analysis. By 2020, oil crop cultivation caused the potential long-term loss of 1.5% of global species (plants and terrestrial vertebrates), with oil palm, coconuts and soybeans responsible for three-quarters of this effect. Tropical regions, occupying just under half of harvested area, bear almost four-fifths of impacts. More than half of this loss was outsourced through international trade, as major importers displace biodiversity burdens to tropical regions. Biodiversity impacts increased by four-fifths since 1995, statistically attributed primarily to rising per capita consumption. Our findings identify production hotspots and consumption-side contributions, supporting targeted interventions to mitigate biodiversity impacts along oil crop supply chains.

## Abstract (web summary)

Spatial assessments reveal that oil crop expansion, particularly in tropical regions, threaten global biodiversity. Hotspots of production and consumption are identified, showing that consumption-side interventions can aid in developing strategies to reduce biodiversity impacts.

## Main

Biodiversity loss is one of the most pressing global challenges, threatening ecosystem services, food security and climate resilience and is largely driven by agricultural land use (that is, land occupation, land transformation and intensification). The global oil crop cultivation is a major contributor to ongoing land-use changes and has thus emerged as a critical driver of biodiversity loss. For instance, oil palm cultivation replacing natural habitats in Southeast Asia causes substantial deforestation and species extinction^1–3^. By 2020, oil crops occupied approximately 330 million hectares, representing 22% of the world’s cropland, and their total area had increased by approximately 58% since 1995^4^. Oil crop cultivation is expected to continue expanding in response to growing global demand for vegetable oils, biofuels and other by-products used in the food and non-food industries^5,6^. Although numerous studies have assessed the region-specific biodiversity impacts of individual oil crops such as oil palm^2,5,7^ and soybeans^8,9^, these assessments lack a comprehensive global perspective across all oil crops and their full supply chains.

Due to the globalization of supply chains, biodiversity impacts caused by oil crop cultivation in production regions can be driven by demand from other parts of the world^10–12^. For example, palm oil consumption in the European Union (EU) has led to extensive deforestation in Indonesia, resulting in a substantial increase in local species loss^13^. To trace and quantify these outsourced environmental impacts across the global economy, multi-region input–output (MRIO) analysis is commonly employed^14–17^. However, existing MRIO frameworks face substantial challenges when it comes to accurately capturing the biodiversity impacts of specific oil crops. These challenges stem from coarse sector and regional classifications, incomplete global economic coverage^18–20^ and a lack of detailed, region-specific data on the biodiversity impacts of various crops. Moreover, classic MRIO analysis typically focuses on the direct links between production and consumption, overlooking the intermediate stages within the supply chain^18^. As a result, although classic MRIO analysis can identify the final consumer countries and products such as cosmetics that ultimately drive oil palm cultivation, it fails to provide detailed insights into how biodiversity impacts are transferred at each stage, from oil palm cultivation and palm oil refining to the final consumer goods.

Here we address the above knowledge gaps by (1) quantifying spatially explicit biodiversity impacts at grid cell level (0.05°) to identify hotspot areas (grid cells with high impact) and critical regions (countries or regions with disproportionately high contributions to biodiversity impacts) linked to oil crop cultivation, (2) tracking biodiversity impacts along the full supply chain from production to consumption (including intermediate processing steps) and (3) decomposing the socio-economic contributors of biodiversity impacts over time. First, we synthesized the global spatial distribution of oil crops in 2020 through the integration of multiple datasets, including Food and Agriculture Organization Corporate Statistical Database (FAOSTAT)^4^, Spatial Production Allocation Model (SPAM)^21^, CROPGRIDS^22^ and remote-sensing-based global crop distribution maps^23,24^. Crop-specific land-use intensity (LUI) was calculated using global nitrogen and phosphorus application datasets^25,26^ and irrigation datasets where applicable^27^. Second, we combined the spatial distribution and LUI of oil crops with global species loss factors (LFs) to calculate potential global species loss (PSLglo). We use PSLglo as a key indicator of biodiversity impacts because it is a robust, globally comparable metric recommended by the United Nations Environment Programme (UNEP) that directly quantifies potential global species extinctions caused by land use^28,29^. Third, we hybridized the food and agriculture biomass input–output model (FABIO)^20^, which has detailed classifications of oil crops and their by-products, with Resolved EXIOBASE3 (REX3) MRIO^19^, which provides detailed classifications of remaining non-food final uses. This hybridized MRIO framework enables comprehensive analysis by providing both detailed sector classification and extensive economic coverage across the global oil crop supply chains. Finally, we linked PSLglo to the hybridized MRIO, applying enhanced supply chain mapping^15,18,30,31^ and structural decomposition analysis (SDA)^32,33^ to systematically track the biodiversity impacts and its socio-economic contributors along oil crop supply chains. Our findings identify hotspot areas and critical regions and reveal the transfer mechanisms of biodiversity loss along global oil crop supply chains, providing insights to guide targeted cross-border policy coordination and responsible sourcing strategies.

## Results

### Hotspot areas and critical regions of biodiversity impact due to oil crop cultivation

**Fig. 1:**
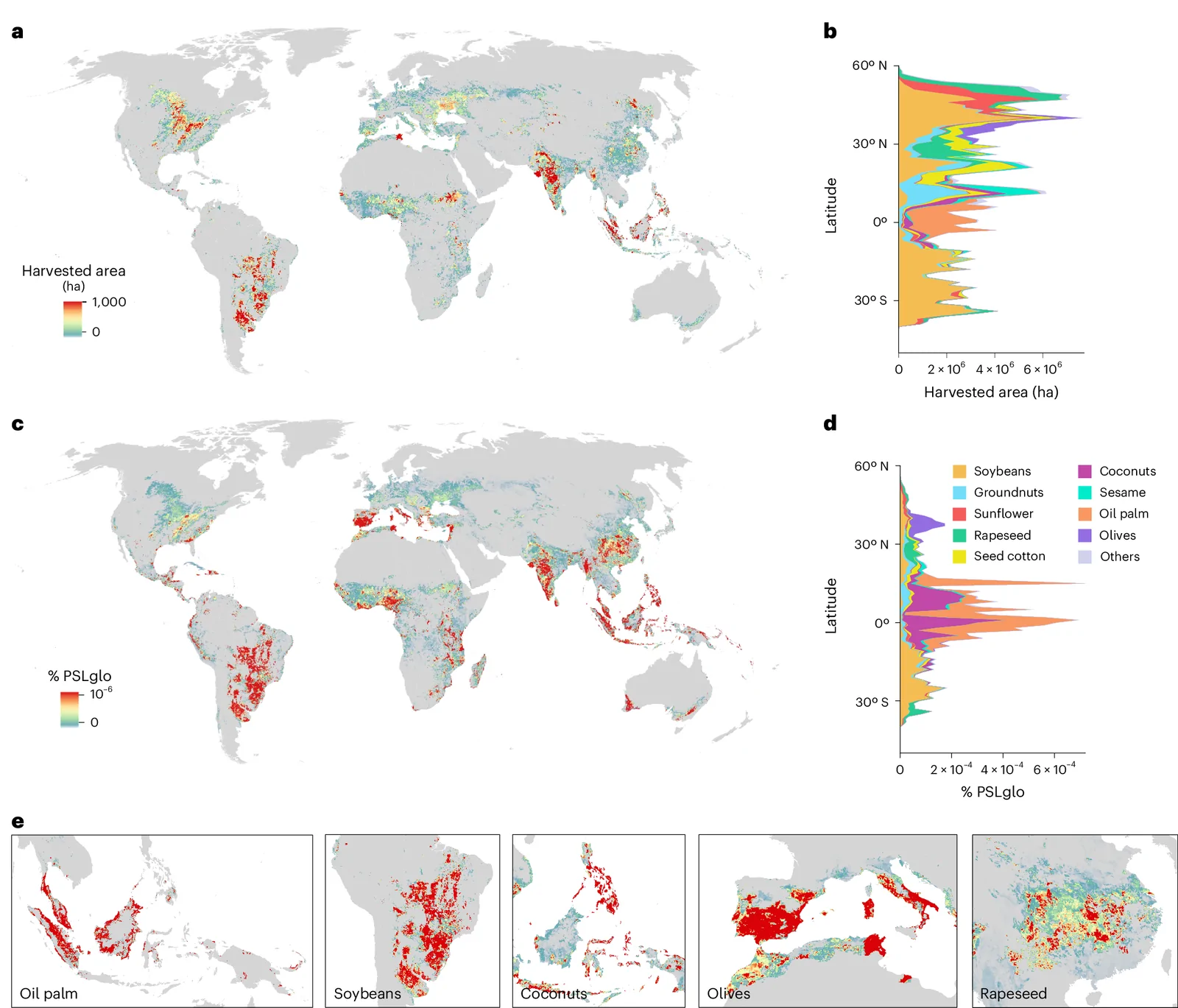
Global spatial distribution of oil crop cultivation and related global species loss. **a**, Global spatial distribution of the harvested area of oil crops in 2020. **b**, Stacked chart of the harvested area of major oil crops with latitudinal variation. **c**, Global spatial distribution of biodiversity impacts from oil crop cultivation in 2020, measured as PSLglo. PSLglo is expressed as a percentage (%), representing the proportion of global species (plants and terrestrial vertebrates) facing potential extinction due to related land use. **d**, Stacked chart of the global PSLglo of major oil crop cultivation with latitude variation. The same legend was used for panels **b** and **d**. **e**, Hotspot areas of global species loss from major oil crop cultivation, defined as grid cells with high biodiversity impact (>10^−6^% PSLglo), corresponding to approximately the 90th percentile (top 10%) of impacted grid cells. The same colour scale is used for panels **c** and **e**. All results are presented at 0.05° spatial resolution (approximately 5.5 km at the equator), with harvested area and PSLglo values calculated for each individual grid cell. Source data

Figure 1a,b illustrates the global harvested area of oil crop cultivation. Soybeans account for approximately 39% of the total harvested area, followed by rapeseed, seed cotton, groundnuts, oil palm and sunflower (each representing 8% to 10%), whereas coconuts, together with sesame, olives and other minor oil crops, collectively occupy the remaining share. The spatial patterns are heavily influenced by regional climate and latitude. For instance, soybeans are mainly grown in the temperate or subtropical regions of North and South America, whereas oil palm and coconuts remain largely confined to the tropical zones of Southeast Asia. Such regional differences in key cultivation areas result in considerable variations regarding biodiversity impact.

Oil crop cultivation drives substantial biodiversity impacts, reaching a PSLglo of about 1.52% in 2020, indicating that the associated land use has generated a global extinction risk equivalent to the potential loss of 1.52% of global terrestrial species (plants and terrestrial vertebrates). However, these impacts are highly concentrated: tropical regions (23.5° S–23.5° N), which represent only 43% of the harvested area, account for a disproportionate 78% of the total biodiversity impacts (Fig. 1c,d). Among individual crops, oil palm occupies about 9% of the harvested area yet contributes approximately 34% of total PSLglo, whereas coconuts, covering less than 4% area, account for about 23% PSLglo. By contrast, soybeans, despite claiming 39% of the harvested area, account for only 18% of PSLglo. The disparity also exists within the same oil crops cultivated in different regions. For example, while the USA accounts for 26% of the world’s soybean harvested area, it contributes only 7% of the biodiversity impacts caused by soybean cultivation, whereas Brazil, with 29% share of harvested area, is responsible for a disproportionate 60% of the associated biodiversity impacts. Figure 1e identifies hotspot areas, defined as grid cells with high biodiversity impact (>10^−6^% PSLglo), corresponding to approximately the 90th percentile (top 10%) of impacted grid cells. These hotspots reveal critical regions with disproportionately high contributions to global species loss, including oil palm and coconut cultivation in Southeast Asia (notably Indonesia, Malaysia and the Philippines), soybean production in South America (especially Brazil), olive cultivation in Mediterranean countries (for example, Spain, Italy, Tunisia) and rapeseed cultivation in China.

### Biodiversity impacts embodied in shifting oil crop supply chains

**Fig. 2:**
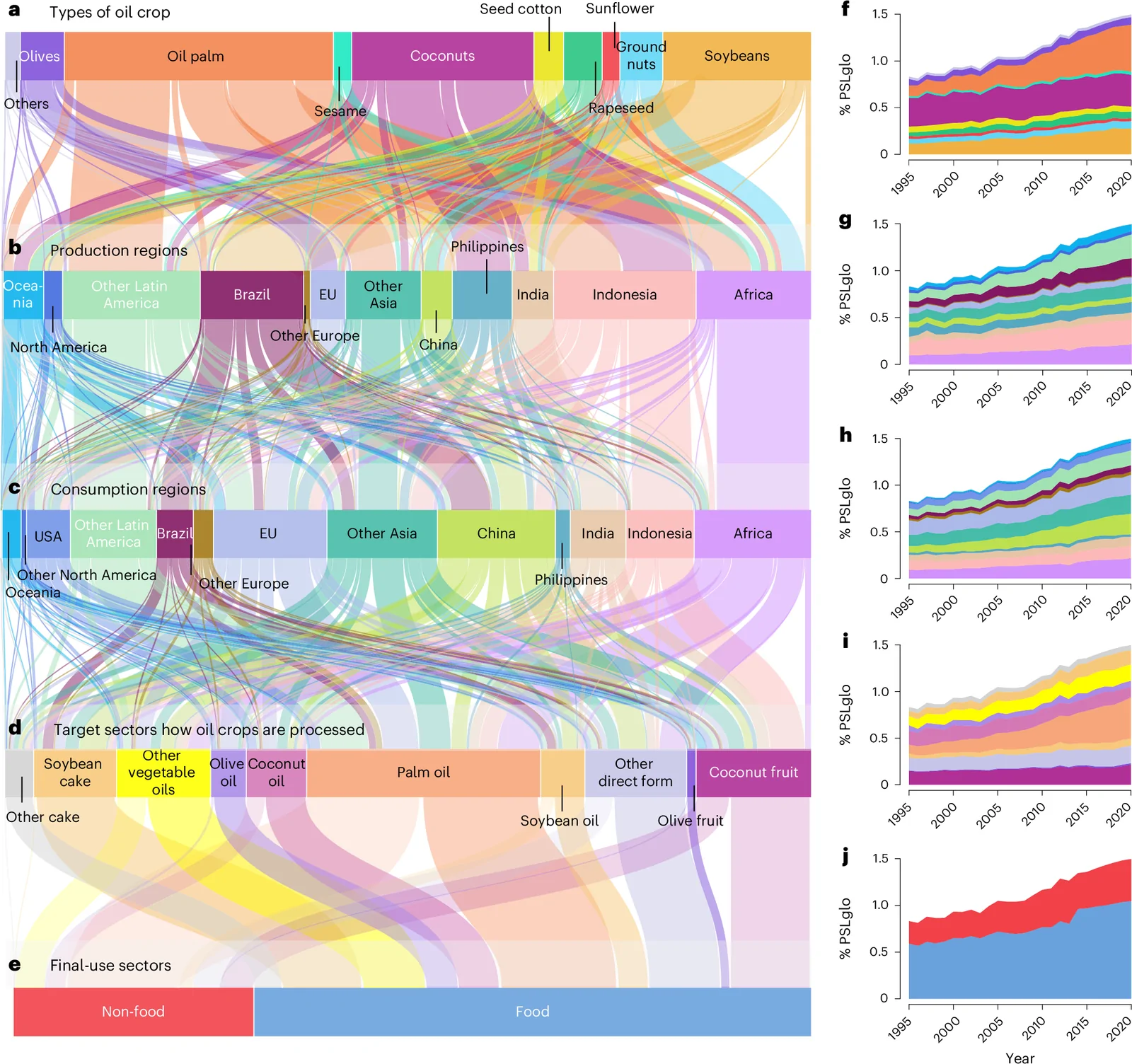
Global supply chain analysis of oil crops and their biodiversity impacts from 1995 to 2020. **a**–**e**, Sankey diagram showing the interconnections of oil crop supply chains in 2020 from five perspectives: types of oil crop cultivated (**a**), production regions where crops are cultivated (**b**), consumption regions (**c**), intermediate product sectors (**d**)—minor sectors are aggregated for clarity (Supplementary Table 3 provides the full sector list and mapping)—and final-use sectors (**e**). The width of the flows represents the magnitude of biodiversity impact under the land-use conditions in 2020, measured as potential global species loss (PSLglo). PSLglo is expressed as a percentage (%), representing the proportion of global species (plants and terrestrial vertebrates) facing potential extinction risk due to related land use. **f**–**j**, Temporal evolution of biodiversity impacts from 1995 to 2020 corresponding to the five perspectives shown in the Sankey diagram: by oil crop types (**f**), by production regions (**g**), by consumption regions (**h**), by target/intermediate processing sectors (**i**) and final-use sectors (**j**). All panels are based on our enhanced supply chain impact mapping that covers most regions with traceable supply chains for food and non-food uses and accounts for 98% of the total biodiversity impacts in 2020. The colour scheme in panels **f**–**j** corresponds to the respective categories in the Sankey diagram in panels **a**–**e**. The *y* axis represents the biodiversity impact measured in percent PSLglo under the land-use conditions of the corresponding year. Source data

Figure 2a–e illustrates the global biodiversity impacts along oil crop supply chains in 2020. Oil palm, coconuts and soybeans together accounted for nearly three-quarters of the total impacts (Fig. 2a). Regarding production regions (Fig. 2b), Asia and Latin America collectively accounted for approximately 75% of these impacts. Asia’s share (approximately 43%) was mainly due to oil palm and coconut cultivation in Indonesia and the Philippines. Latin America’s share (approximately 30%) primarily resulted from soybean cultivation in Brazil and oil palm cultivation in Guatemala and Honduras. From the intermediate product perspective (Fig. 2d), vegetable oils accounted for 58% of total PSLglo, with palm oil alone contributing 29%. Oil cake production, predominantly derived from soybeans, represented 14% of the total impact.

**Fig. 3:**
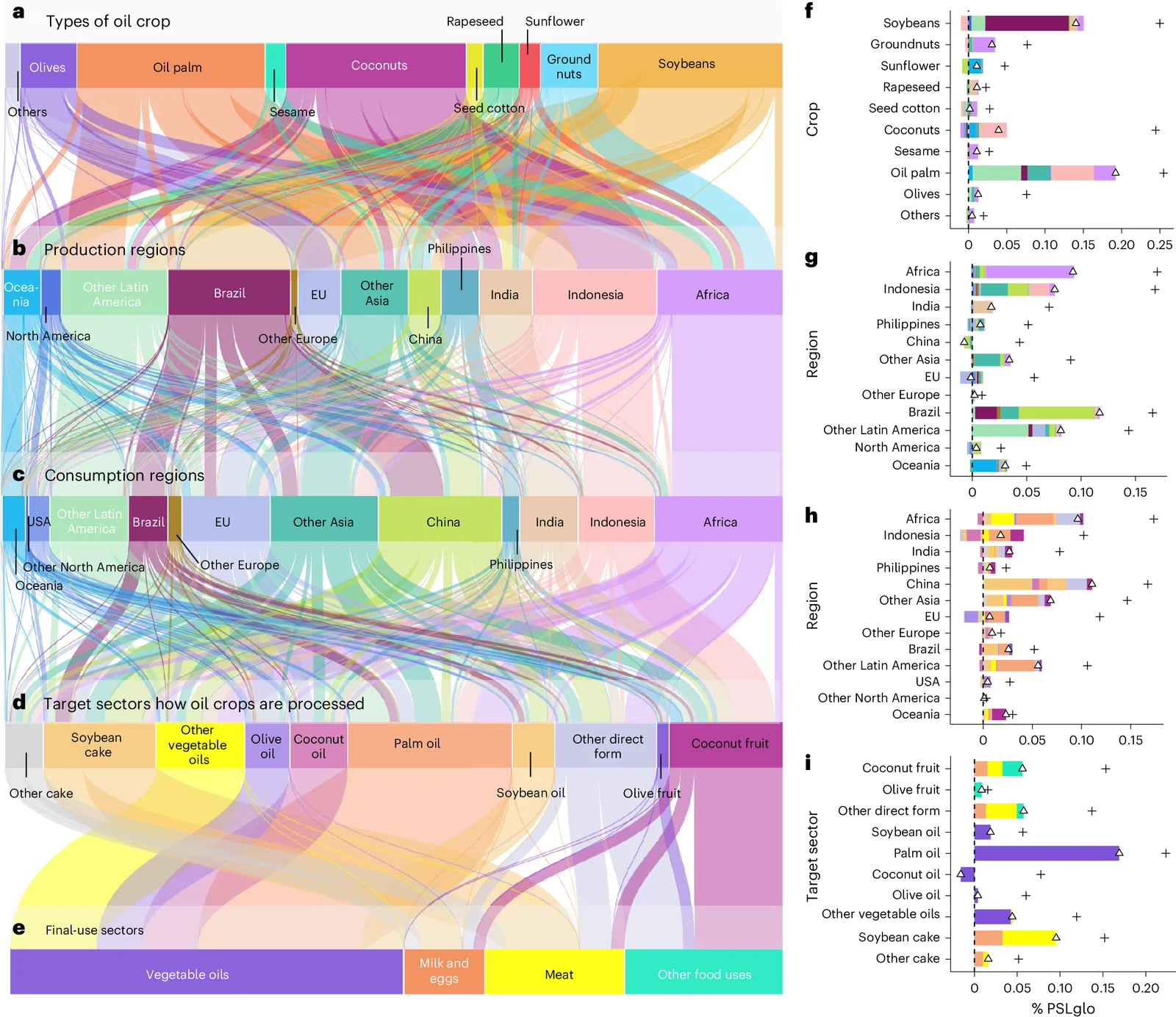
Global supply chain analysis of oil crops and their biodiversity impact for food final uses from 1995 to 2020. **a**–**e**, Sankey diagram illustrating the interconnections of oil crop supply chains in 2020 from five perspectives: types of oil crop cultivated (**a**), production regions where crops are cultivated (**b**), consumption regions (**c**), target/intermediate processing sectors (**d**)—minor sectors are aggregated for clarity (Supplementary Table 3 provides the full sector list and mapping)—and final-use sectors (including vegetable oils, milk and eggs, meat, and other food uses) (**e**). The width of the flows represents the magnitude of biodiversity impact measured in PSLglo. PSLglo is expressed as a percentage (%), representing the proportion of global species (plants and terrestrial vertebrates) facing potential extinction due to related land use. **f**–**i**, Temporal analysis of biodiversity impact shifts from 1995 to 2020 is presented through stacked bar charts showing: changes in biodiversity impact by oil crop type (**f**), with bars showing distribution across production region, changes by production region (**g**), with bars showing distribution across consumption region, changes by consumption region (**h**), with bars showing distribution across intermediate processing sectors, and changes by intermediate processing sectors (**i**), with bars showing distribution across final-use sectors. The colour scheme in the stacked bar charts corresponds to the respective categories in the Sankey diagram for each supply chain perspective. In all stacked bar charts, the horizontal axis represents biodiversity impact magnitude (% PSLglo), hollow triangles indicate the total net change in biodiversity impact from 1995 to 2020 and plus signs denote the total biodiversity impact in 2020. Source data

**Fig. 4:**
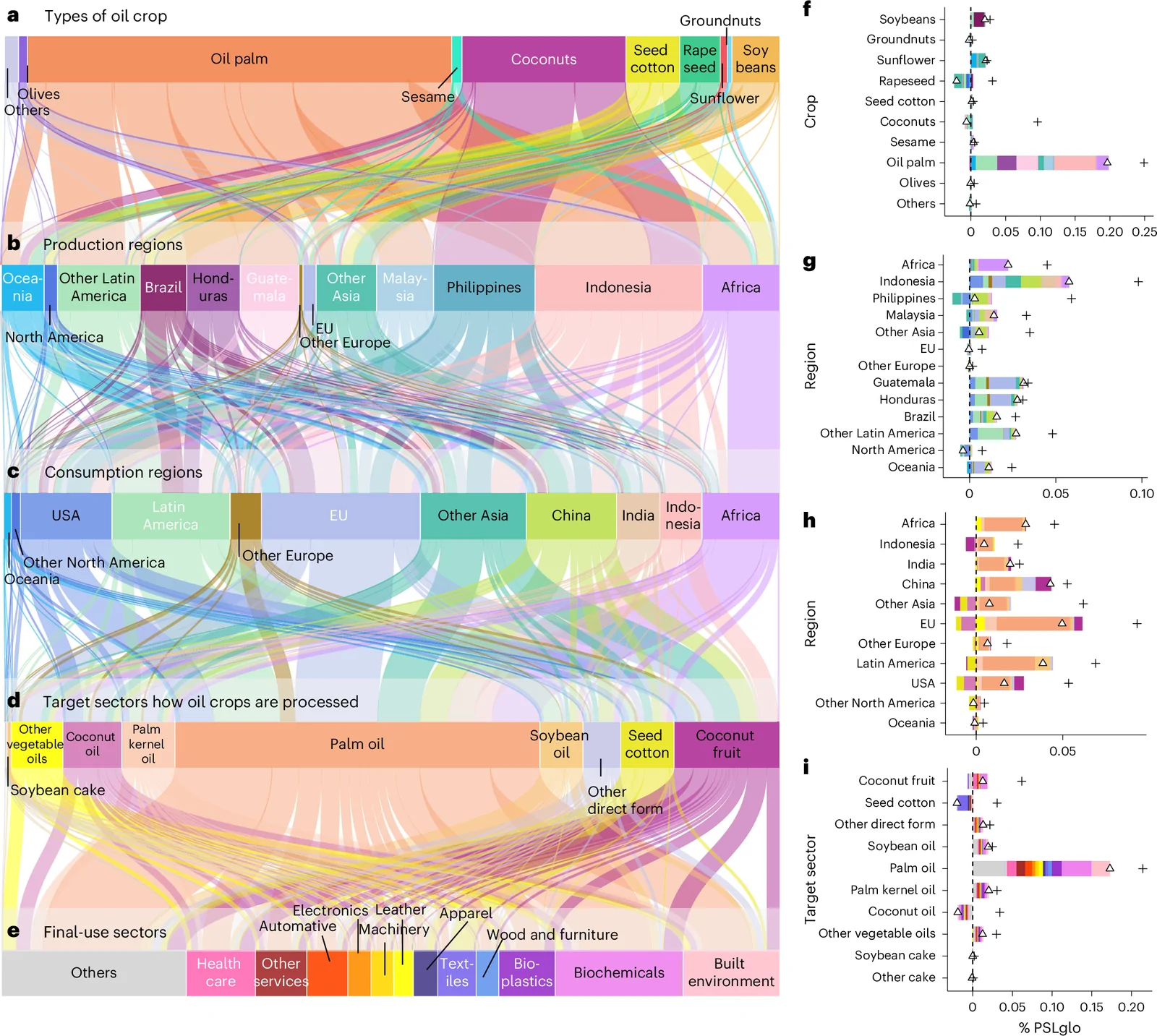
Global supply chain analysis of oil crops and their biodiversity impact for non-food final uses from 1995 to 2020. **a**–**e**, Sankey diagram illustrating the interconnections of oil crop supply chains in 2020 from five perspectives: types of oil crop cultivated (**a**), production regions where crops are cultivated (**b**), consumption regions (**c**), target/intermediate processing sectors (**d**)—minor sectors are aggregated for clarity (Supplementary Table 3 provides the full sector list and mapping)—and final-use sectors (**e**). The width of the flows represents the magnitude of biodiversity impact measured in PSLglo. PSLglo is expressed as a percentage (%), representing the proportion of global species (plants and terrestrial vertebrates) facing potential extinction due to related land use. **f**–**i**, Temporal analysis of biodiversity impact shifts from 1995 to 2020 is presented through stacked bar charts showing: changes in biodiversity impact by oil crop type (**f**), with bars showing distribution across production region, changes by production region (**g**), with bars showing distribution across consumption region, changes by consumption region (**h**), with bars showing distribution across intermediate processing sectors, and changes by intermediate processing sectors (**i**), with bars showing distribution across final-use sectors. The colour scheme in the stacked bar charts corresponds to the respective categories in the Sankey diagram for each supply chain perspective. In all stacked bar charts, the horizontal axis represents biodiversity impact magnitude (% PSLglo), hollow triangles indicate the total net change in biodiversity impact from 1995 to 2020 and plus signs denote the total biodiversity impact in 2020. Source data

Food final uses accounted for approximately 70% of biodiversity impacts, whereas non-food final uses accounted for approximately 30% (Fig. 2e). Within food final uses (Fig. 3), direct vegetable oil consumption accounted for approximately 51% of the total PSLglo for food final uses. Animal products contributed an additional 29%, primarily driven by soybean cake used as feed in meat, dairy and egg production. Direct human consumption of olives, coconut fruits and other edible oil crop products was responsible for the remaining 20%. Within non-food final uses (Fig. 4), the EU was responsible for the largest share of impacts (approximately 21%), followed by Latin America (15%), the USA (12%) and China (12%). Vegetable oils contributed nearly three-quarters of PSLglo for non-food final uses. These impacts were distributed across multiple industrial applications. Biochemicals (including biodiesel) accounted for the largest share (approximately 17%), followed by the built environment (13%), healthcare (9%) and bioplastics (7%). The EU accounted for approximately 63% of the PSLglo associated with bioplastics applications. Detailed analyses of biodiversity impacts for food and non-food final uses are provided in Supplementary Results 1 and 2.

Figure 2f–j illustrates that global biodiversity impacts associated with oil crop cultivation increased by approximately 80% (0.67% PSLglo) from 1995 to 2020 (relative to the 1995 baseline, covering most regions with traceable supply chains for food and non-food final uses). According to the planetary boundaries (PB) framework, biosphere integrity has been identified as a core PB that is already transgressed globally^34–36^. Recent studies have proposed using the annual change rate of PSLglo as an alternative indicator for tracking biosphere integrity PB, with a suggested threshold of 0.001% PSLglo per year (refs. ^37–39^). Our findings show that oil crop cultivation alone contributed an annual species loss rate that strongly surpassed this threshold, indicating a substantial contribution of oil crop expansion to the transgression of the biosphere integrity PB. This PSLglo increase was predominantly driven by rising impacts from oil palm and soybeans. Oil palm alone accounted for 60% of the total increase in biodiversity impacts, with Latin America, Indonesia and other regions contributing 25%, 18% and 17%, respectively. Soybean cultivation contributed an additional 25%, primarily due to expansion in Brazil (21%). From the production regions perspective, rapid biodiversity impact increases occurred predominantly in tropical regions such as Southeast Asia and Latin America, whereas impacts in temperate regions (for example, USA, EU and China) showed minimal growth (<4%). From the consumption regions perspective, developing economies drove most of the global increase: China’s biodiversity impacts rose approximately 3.3-fold, Africa’s increased by 2.3-fold and Latin America’s by 2.3-fold (all relative to 1995). Notably, China’s growth was largely driven by imports, whereas Africa’s increase predominantly resulted from expanding domestic production.

From the intermediate product perspective, increased demand for vegetable oils contributed the most, accounting for over 64% of the overall growth in PSLglo. Palm oil alone represented approximately 51% of this increase. Coconut oil was the only vegetable oil category showing a decline in biodiversity impacts; however, this reduction was offset by substantial growth from other vegetable oils. Among non-vegetable oil intermediate products, soybean cake made the largest contribution to the total increase (approximately 14%), followed by coconut fruits (10%).

From a final-use perspective, global biodiversity impacts associated with food consumption rose by approximately 77% between 1995 and 2020, whereas those associated with non-food consumption nearly doubled (+89%). Growth was most pronounced in China, where food-related impacts increased by 196% and non-food-related impacts increased by 438%. In contrast, the EU showed a different pattern: food-related impacts rose only marginally (+6%), whereas non-food impacts more than doubled (+115%), largely fuelled by increased consumption of bio-based materials. Whereas the EU’s shift towards renewable bio-based energy and materials has contributed to reducing carbon emissions, it has simultaneously intensified biodiversity loss by driving increased demand for oil crops cultivated in biodiversity-rich tropical regions.

### Outsourced biodiversity impacts along the global oil crop supply chains

International trade played a critical role in displacing biodiversity impacts from production to consumption regions along global oil crop supply chains. In 2020, the outsourced share of total impacts reached 54% (Fig. 2b,c). Outsourcing shares differed substantially across food and non-food consumption: biodiversity impacts associated with food final uses showed an outsourced share of 42% (Fig. 3b,c), whereas impacts related to non-food final uses reached as high as 79% (Fig. 4b,c). Three major importing regions exhibited notably high proportions of outsourcing: China outsourced 77% of its consumption-based impacts, the EU outsourced 79% and North America outsourced 86%. Collectively, these three regions contributed nearly 60% to global outsourced biodiversity impacts, with China and the EU each accounting for 24% and North America for 12%.

The outsourced biodiversity impacts fell disproportionately upon biodiversity-rich tropical production regions. In Southeast Asia, Indonesia (21%) and the Philippines (13%) bore substantial portions of these outsourced impacts, while in Latin America, Brazil alone accounted for 20% of global outsourced biodiversity impacts. In total, these tropical regions absorbed approximately 70% of globally outsourced biodiversity impacts. Specifically, China’s outsourced biodiversity impacts were primarily driven by soybean imports from Brazil, whereas the EU’s outsourced impacts largely stemmed from tropical oil crops, particularly palm oil imported from Southeast Asian countries such as Indonesia. Consequently, international trade has facilitated a substantial transfer of biodiversity impacts from temperate consumption regions to tropical production regions, thereby increasing ecological pressures in these vulnerable regions.

Between 1995 and 2020, biodiversity impacts outsourced via international trade increased approximately 2.4-fold (a 133% increase), accounting for roughly two-thirds (68%) of the total global increase in biodiversity impacts along oil crop supply chains. When categorized by final use, outsourced biodiversity impacts represented 58% of the biodiversity impact growth attributed to food final uses, whereas this proportion reached 91% for non-food final uses. Among consumption regions, China’s growing demand emerged as one of the major drivers of outsourced biodiversity impacts, increasing nearly 11-fold and accounting for 34% of the global growth in outsourced impacts. The EU was the second largest contributor, with an 87% increase, contributing 17% to global growth. Taken together, these large consumption regions in both emerging (for example, China) and developed economies (for example, EU) jointly drive the observed rise in outsourced biodiversity impacts along global oil crop supply chains.

### Per capita consumption accounts for the largest share of biodiversity impact growth

**Fig. 5:**
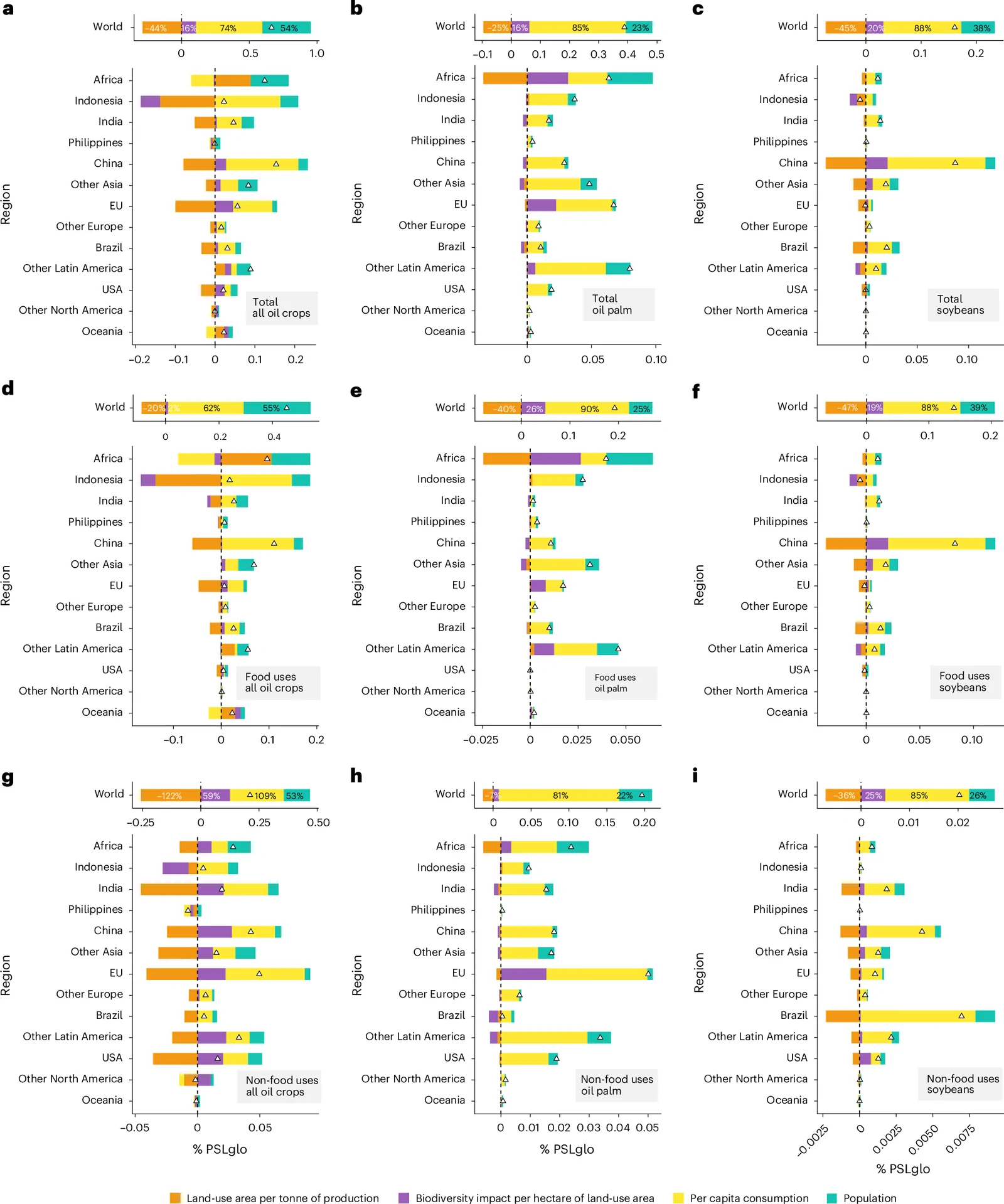
SDA of changes in biodiversity impacts from oil crop cultivation across global regions from 1995 to 2020. **a**–**c**, The figure shows stacked bar charts illustrating the contribution of four key decomposition factors: biodiversity impacts per hectare of land-use area (purple), land-use area per tonne of production (orange), per capita consumption (yellow) and population growth (turquoise). Bar charts are organized in three columns, representing total impacts for all crops (**a**), total oil palm (**b**) and total soybeans (**c**), respectively. **d**–**i**, Similar analysis for food final uses (**d**–**f**) and results for non-food final uses (**g**–**i**). Each row displays data for different world regions including Africa, Indonesia, India, Philippines, China, other Asia, EU, other Europe, Brazil, other Latin America, USA, other North America and Oceania. The horizontal axis represents the magnitude of impact change (% PSLglo). PSLglo represents the proportion of global species (plants and terrestrial vertebrates) facing potential extinction due to related land use. Triangle markers (Δ) indicate the net change in biodiversity impacts, calculated as the sum of all decomposition factors. The length and direction of bars indicate the relative contribution of each decomposition factor, with positive values (right of zero) showing factors that increased biodiversity impacts and negative values (left of zero) showing factors that decreased impacts. The percentage values shown above the world bars represent each factor’s proportional contribution to the total change in biodiversity impacts. Percentages of decomposition factors may exceed 100% when positive and negative contributions offset each other. Source data

Using SDA, we statistically attribute the observed increase in biodiversity impacts from global oil crop supply chains between 1995 and 2020 primarily to changes in per capita consumption, which accounted for a contribution equivalent to ~74% of the total net increase (Fig. 5a). Population growth was the second largest contributor (~54%). These contributions were partly offset by a negative contribution from reduced land-use area per tonne of production of approximately −44%, consistent with yield improvements and shifts in the oil crop mix towards higher-yielding crops. However, the analysis also indicates that agricultural expansion increasingly occurred in biodiversity-rich regions, reflected in a 16% contribution from the biodiversity impacts per hectare of land-use area.

Regionally, China emerged as the country where rising per capita consumption made the largest contribution to the increase in consumption-based biodiversity impacts. Between 1995 and 2020, China’s per capita consumption nearly quadrupled and thus contributed 119% of the net increase in its biodiversity impact. Other emerging economies, including India, Brazil and Indonesia, also exhibited considerable growth in biodiversity impacts, statistically attributed primarily to increased per capita consumption. By contrast, per capita consumption contributed less to the net increase in most developed economies over the same period. Nevertheless, despite rapid consumption growth in emerging regions, biodiversity impacts per capita in developing economies remain substantially lower than those in developed regions such as the EU and the USA; for example, in 2020, China’s consumption-based biodiversity impact per capita from the global oil crop supply chain was still about 63% lower than that of the EU. In Africa, SDA assigns a substantial positive contribution to increases in land-use area per tonne of production, reflecting declining oil crop agricultural efficiency. Our supply chain analysis indicates that more than 80% of Africa’s oil crop consumption is locally sourced, suggesting that investing in and adopting sustainable agricultural practices and improved farming methods would be particularly relevant in Africa. Except for Africa, most regions experienced reductions in land-use area per tonne of production due to improvements in agricultural practices and the adoption of more efficient crop varieties. However, increased reliance on high-yield oil crop cultivation (for example, oil palm), often located in biodiversity-rich regions, has geographically shifted biodiversity impacts. A more detailed analysis of the SDA results is provided in Supplementary Results 3.

## Discussion

Our study integrates spatially explicit biodiversity impact assessments that explicitly incorporate LUI with a hybrid MRIO framework and detailed supply chain impact mapping. These methodological improvements, particularly the differentiation of LUI levels and the application of spatially explicit LFs, result in biodiversity impact estimates for global oil crop cultivation of 1.52% PSLglo in 2020, almost three times higher than previously reported (0.56% PSLglo)^39^, which did not account for the spatial distribution of oil crops and variation in LUI and therefore substantially underestimated their biodiversity impacts. However, to enable a consistent global comparison, our framework necessarily simplifies (1) the spatio-temporal representation of crop expansion and land-use change (including the focus on land occupation rather than explicit land transformation), (2) biodiversity responses through generalized land-use classes and ecoregion‑average loss factors and (3) certain supply chain and sectoral distinctions (for example, biofuels). Whereas our SDA results identify the relative contributions of consumption and efficiency, it is important to note that SDA is a mathematical accounting framework rather than a causal model. It treats factors such as per capita consumption and technical efficiency as independent, whereas in reality, these variables are often interdependent. For example, improvements in agricultural efficiency can lower production costs and prices, which may subsequently stimulate higher demand through the rebound effect. Consequently, these results should be viewed as a statistical attribution of historical trends rather than a prediction of how these variables would interact in a dynamic economic system. We discuss these considerations and limitations in Supplementary Discussion 1 and outline priorities for future work. Looking ahead, integrating temporally explicit land‑use change and more granular supply chain information and complementing the endpoint footprint with finer‑resolution, spatially explicit biodiversity indicators (linked to globally comparable endpoint metrics)^40,41^, could further improve the accuracy and decision relevance of biodiversity impact assessment of global agri‑food supply chain analysis.

Our findings offer critical policy insights to mitigate the biodiversity impacts of oil crop cultivation and promote more sustainable production and consumption patterns. Sustainable cultivation in pan-tropical regions, notably Southeast Asia and Latin America, is vital given their disproportionate biodiversity impacts despite modest harvested area shares. There is an immediate need for more stringent deforestation restrictions^42,43^ and the reinforcement of certification systems^44^ such as the Roundtable on Sustainable Palm Oil (RSPO)^45^ to mitigate these impacts.

Beyond production hotspots, our analysis shows that major importing economies, particularly China and the EU, account for nearly half of the global outsourced biodiversity impacts along global oil crop supply chains. This highlights the need for these regions to promote sustainable sourcing through increased consumer awareness^46,47^, stricter import standards^48^ and the creation of incentives for responsible suppliers^49^. Such measures can drive shifts towards sustainable dietary patterns via demand reduction. However, their effectiveness depends on broader market feedback and price-demand elasticities not explicitly modelled in our accounting framework. Meanwhile, dietary shifts in the global south also require attention, as improved nutrition driven by higher consumption of vegetable oils and animal products has inadvertently increased biodiversity impacts. Therefore, policy interventions should address the environmental implications of dietary transitions while ensuring nutritional security.

Furthermore, policy focus should differ for food and non-food final uses. Food consumption is the main driver of current biodiversity impacts (~70%), primarily from vegetable oils and animal feed, requiring policies that promote sustainable diets while balancing global nutritional needs. Non-food uses (~30%), though smaller currently, are growing faster (+89%) and rely heavily on trade outsourcing (82%). Their drivers (for example, biochemicals, bioplastics) are often linked to climate objectives. This requires a careful assessment of the environmental footprint of bio-based alternatives to ensure alignment between climate and biodiversity policies, particularly regarding imported feedstocks.

Moreover, robust international supply chain traceability systems are fundamental to ensuring environmental compliance across borders. Enhanced transparency in oil palm and soybean supply chains (potentially leveraging technologies such as blockchain) could substantially improve the monitoring and enforcement of sustainability standards.

Finally, our results indicate that decreases in land-use area per tonne of production (reflecting yield gains within specific crops and shifts towards higher-yielding crops) substantially counteracted increased consumption-side contribution to biodiversity impacts over 1995–2020. This indicates that agricultural productivity improvement served as a critical leverage point historically. Moving forward, policy efforts might benefit from targeted investments in agricultural research and technological innovations focused on sustainably intensifying production on existing farmland. However, achieving this without triggering rebound effects requires complementary measures to ensure that yield gains genuinely alleviate pressure for expansion into biodiversity-rich habitats, rather than inadvertently stimulating new demand, as highlighted in the land-sharing/sparing debate^50^.

In summary, addressing biodiversity impacts from global oil crop cultivation requires integrated policies targeting sustainable production, responsible consumption and enhanced supply chain transparency, while accounting for potential feedback loops and rebound effects. Future research should focus on improving data collection, analytical methods and evidence-based assessments to inform integrated strategies addressing production efficiency, biodiversity conservation and consumer behaviour. Such an approach will support policymakers in promoting sustainable and ecologically responsible oil crop supply chains.

## Methods

### Harmonized global spatial distribution of oil crop cultivation

To quantitatively assess biodiversity impacts associated with global oil crop cultivation, we constructed a detailed spatial distribution map covering 19 major oil crops (Supplementary Table 1). Given the importance of reliable datasets for accurate assessment, we prioritized data sources by reliability, using direct observational data (for example, remote sensing) where available, followed by indirect observational data and land-use statistics. Specifically, high-resolution remote sensing data provided distributions for closed-canopy coconuts^23^ and oil palm plantations^24^, whereas the global spatially disaggregated crop production statistics data for 2020 (SPAM 2020)^21^ provided distributions for coconuts, soybeans, groundnuts, sunflower, rapeseed, sesame and seed cotton. For the remaining oil crops, we used CROPGRIDS^22^ data, a global gridded dataset providing spatially explicit distribution for numerous crops.

To harmonize the diverse input datasets, we first resampled all spatial layers to a uniform 0.05° spatial resolution (approximately 5.5 km at the equator), consistent with CROPGRIDS. Subsequently, we calibrated our preliminary maps to align with the national harvested area statistics reported by FAOSTAT (2020). This calibration involved three steps: (1) aggregating our preliminary, pre-calibration mapped harvested area estimates to the country level using standardized administrative boundaries from Natural Earth^51^; (2) deriving country-specific calibration factors, calculated as the ratio of FAOSTAT-reported national harvested area to our aggregated harvested area estimates; and (3) systematically applying these calibration factors back to the original harvested and cultivated area maps, thereby adjusting their absolute magnitudes within each country while preserving their inherent relative spatial distribution patterns. Harvested area refers to the total area from which crops are gathered annually, potentially including multiple harvests per year on the same land. In contrast, cultivated area represents the physical extent of land dedicated to crop production, encompassing areas including fallow and rotated plots, which better reflects the annual land occupation. Although the calibration factors were derived from harvested area discrepancies, we applied them to cultivated area maps under the assumption that any systematic country-level bias affects both metrics similarly. This methodological workflow resulted in a globally harmonized spatial distribution for oil crop cultivation in 2020, serving as the spatial baseline for our temporal reconstruction.

To enable temporal analysis, we reconstructed annual historical distributions of oil crop cultivated area (1995–2019) by combining the 2020 maps with the HYDE 3.5 historical cropland dataset^52^, following the method of Mialyk et al.^53^. Specifically, for each 0.05° grid cell, we calculated the fraction of each oil crop relative to the total HYDE cropland in 2020. In cells where total oil crop area exceeded reported HYDE cropland, we instead calculated the share relative to the total oil crop cultivated area. We then applied these 2020-derived fractions to annual HYDE maps to scale oil crop cultivated areas proportionally to historical cropland changes (truncating negative values to zero). This procedure produced a consistent set of annual maps at 0.05° resolution. These datasets assume that the within-grid crop mix remains constant at 2020 patterns while total cropland area evolves according to HYDE 3.5 data, providing the basis for the temporal biodiversity impact assessment.

### Spatially explicit biodiversity impact assessment of global oil crop cultivation

To quantify biodiversity impacts due to oil crop cultivation, we used state-of-the-art global species loss factors (LFs) developed by Scherer et al.^29^. These LFs cover plants and terrestrial vertebrates (amphibians, birds, mammals and reptiles) and offer a more refined and updated assessment than the benchmark LFs currently recommended by UNEP^28^. By incorporating land-use intensity (LUI) and habitat fragmentation, the LFs estimate the per hectare global potential species loss (PSLglo) due to land occupation at the level of terrestrial ecoregions. The LFs integrate a global extinction probability weighting, which scales local species losses according to each species’ global range and degree of endemism. These per hectare PSLglo values indicate the potential for long-term global species loss resulting from ongoing land-use activities^28,29^. These losses extend beyond immediate extinctions to account for extinction debt due to habitat degradation and fragmentation^28,29^, with the metric ranging from 0 to 1 (here expressed as 0–100%, where 1 or 100% indicates complete extinction risk for all terrestrial species). The LFs provide spatially explicit factors across five broad land-use types (cropland, pasture, plantations, managed forests and urban land) at three intensity levels (minimal, light and intense). For this analysis, we applied the aggregated LFs for plants and terrestrial vertebrates as provided by Scherer et al. These LFs were calculated by first averaging the four terrestrial vertebrates (amphibians, birds, mammals and reptiles) to obtain a kingdom-level factor for Animalia, then averaging this with the plant kingdom factor for each land-use type and intensity level to represent the eukaryotic domain^29^. This aggregation yields an integrated assessment of biodiversity impacts across plants and terrestrial vertebrates, capturing their combined response to land use.

We classified each oil crop according to its corresponding land-use type: tree crops (oil palm, coconuts, olives and so on) were assigned to plantations, whereas annual crops (soybeans, rapeseeds, sunflower, groundnuts and so on) were assigned to cropland. Detailed mappings for all 19 oil crops are provided in Supplementary Table 1. We then calculated the spatial and temporal distribution of LUI for the different oil crops from 1995 to 2020. For tree crops, the industrial oil palm plantations were classified as intense land use, whereas smallholder plantations were classified as light land use. Closed-canopy coconut plantations were categorized as intense land use, whereas other coconut plantations were classified as light land use. For oil palm and coconuts, we computed the within-pixel shares of area under intense versus light LUI based on the 2020 distribution and assumed that these relative shares remain constant over 1995–2019, whereas the total cultivated area per pixel evolves over time. For major annual oil crops with crop-specific nitrogen fertilizer information (soybeans, rapeseeds, sunflower, groundnuts and sesame), we classified LUI based on crop-specific nitrogen fertilizer application data^25^. Nitrogen use of less than 10 kg ha^−1^ was classified as minimal land use, between 10 and 100 kg ha^−1^ as light land use and more than 100 kg ha^−1^ as intense land use. For all remaining crops, we followed Scherer et al.’s method^29^, to construct a general LUI time series by combining area equipped for irrigation (AEI) with annual maps of nitrogen and phosphorus fertilizer application. AEI data were taken from the global dataset developed by Mehta et al.^27^, which provides snapshots at 5‑year intervals up to 2015. We resampled these maps to the 0.05° grid using an area-preserving procedure, linearly interpolated between the 5-year snapshots and linearly extrapolated from 2015 to 2020 to obtain an annual AEI time series for 1995–2020. Nitrogen and phosphorus application rates were derived from the global crop-specific fertilization dataset compiled by Coello et al.^26^, by aggregating across all crop groups and converting reported P_2_O_5_ to elemental P, before resampling to 0.05° resolution to extract annual maps. Using the 2020 global distributions of AEI, nitrogen and phosphorus application rates, we calculated the first and third quartiles (Q1 and Q3) for each indicator and used these fixed thresholds for all years to ensure temporal comparability of LUI classes and avoid shifts in class boundaries: pixels where all three indicators were ≤ Q1 were classified as minimal land use, pixels where at least one indicator exceeded Q3 were classified as intense land use and all others as light land use. After obtaining the spatial and temporal distribution of oil crop LUI, we multiplied the cultivated area of each oil crop by the corresponding land-use type and LUI specific LFs to derive grid cell level PSLglo attributable to oil crop cultivation for each year from 1995 to 2020.

To identify hotspot areas of biodiversity impacts, we first calculated PSLglo for each 0.05° grid cell. We then classified grid cells according to their PSLglo values and defined hotspot areas as cells with PSLglo exceeding 10^−6^%, which in our dataset corresponds approximately to the 90th percentile (top ~10%) of impacted grid cells (PSLglo > 0). These hotspots represent areas with high biodiversity impacts from oil crop cultivation. At the regional scale, we further identified critical regions as countries or regions with particularly high biodiversity impacts. Ultimately, the differences in global species loss from land occupation by various oil crops are driven by the combination of their typical geographic distribution (ecoregions/global extinction probability), land-use type and LUI. For instance, oil palm plantations in Southeast Asian tropical rainforest ecoregions typically cause greater biodiversity impacts per hectare than olive groves in Mediterranean ecoregions due to the higher baseline endemic biodiversity of tropical rainforests.

As a robustness test of our dynamic biodiversity impact time series, we additionally applied a simplified, nationally aggregated approach based on FAOSTAT harvested area statistics (fixed-2020)^4^. In contrast to our spatially explicit reconstruction, which allocated national crop areas within countries to grid cells and time‑varying LUI, this additional test reconstructed temporal dynamics solely from national statistics (FAOSTAT) while holding the national‑average impact per harvested hectare for each crop in 2020 constant. First, we computed for each country and oil crop a national average PSLglo per hectare by dividing the total 2020 PSLglo attributable to that crop by its harvested area in 2020. Second, we multiplied these national average LFs by the annually reported harvested areas from FAOSTAT to obtain country‑ and crop‑specific biodiversity impact time series for 1995–2020. Third, for countries where FAOSTAT-reported harvested areas for a given oil crop, but no spatial distribution information was available, we used the national average LFs of the corresponding land‑use type (cropland or plantation) as a proxy. Comparing this nationally aggregated reconstruction with the dynamic spatially explicit estimates allowed us to assess the robustness of global and regional temporal trends to assumptions about within‑country spatial allocation of oil crops and LUI changes. The relevant comparisons are provided in Supplementary Results 4.

### Hybridized FABIO and REX3

To analyse biodiversity impacts embodied in global oil crop supply chains, we developed a hybrid MRIO model^14,16^ combining the FABIO^20^ and REX3 databases^19,30^, covering the period 1995–2020. FABIO v1.2 serves as our foundation, providing balanced physical MRIO tables covering 192 countries and 123 agricultural and food products from 1986 to 2020. FABIO offers detailed sectoral resolution, including 10 primary oil crop cultivation sectors, 11 vegetable oil sectors and 9 oil cake sectors but lacks coverage of non-food sectors. REX3, a high-resolution global MRIO database derived from EXIOBASE3 and enhanced with detailed country-level data from Eora26 and other international statistics^19,30^, covers 189 countries and 163 sectors for the period 1995 to 2022. REX3 is specifically designed to improve the spatial resolution in regions previously aggregated as ‘Rest of the World’, thereby offering more accurate assessments of environmental impacts linked to global supply chains. We selected REX3 for the hybridized MRIO with FABIO, primarily due to its finer sectoral and spatial resolution and its close alignment with FABIO’s country-level granularity. The hybridized FABIO and REX3 enables comprehensive tracking of oil crops and their by-products from production through intermediate processing to final consumption.

The hybrid MRIO model^14,16,54^ consists of four quadrants: (1) the physical MRIO table of FABIO, (2) the FABIO-to-REX3 linkage matrix, (3) the REX3-to-FABIO linkage matrix and (4) the monetary MRIO table of REX3. While the third quadrant is zero, assuming no downstream linkages from REX3 to FABIO, we mapped the flows of agricultural commodities from FABIO to the regions and sectors of REX3 in the second quadrant of the integrated hybrid MRIO model. We constructed the FABIO-to-REX3 linkage matrix by distributing FABIO’s final demand category ‘other uses’ across REX3 manufacturing sectors based on use shares from REX3 and using a country concordance matrix (Supplementary Table 2). The monetary transaction matrix was computed using supply and use filter matrices (Supplementary Table 8). We then derived the row-stochastic use-share matrix by normalizing these transactions. For each country, the linkage matrix was constructed by multiplying the use shares by the ‘other uses’ demand. Finally, we assembled the full hybrid MRIO matrix, incorporating both physical and monetary flows and computed the hybrid Leontief inverses matrix via blockwise inversion. Its composition is given by equation (1):

1 $$\begin{array}{l}L=\left(\begin{array}{cc}{({I}_{\mathrm{fabio}}-{A}_{\mathrm{fabio}})}^{-1} & {({I}_{\mathrm{fabio}}-{A}_{\mathrm{fabio}})}^{-1}{A}_{\mathrm{other}}{({I}_{\mathrm{rex}3}-{A}_{\mathrm{rex}3})}^{-1}\\ 0 & {({I}_{\mathrm{rex}3}-{A}_{\mathrm{rex}3})}^{-1}\end{array}\right)\\ \,\,\,\,\,\,=\left(\begin{array}{cc}{L}_{\mathrm{fabio}} & {L}_{{\rm{B}}}\\ 0 & {L}_{\mathrm{rex}3}\end{array}\right)\end{array}$$

where *A*_fabio_ and *A*_rex3_ describe the input–output relationships between economic sectors and countries for FABIO and REX3, respectively. *A*_other_ is the matrix of technical coefficients, describing the physical input of agriculture and food products into REX3 sectors per monetary unit of production, thereby linking FABIO’s agricultural sectors to REX3’s non-food sectors. We assume zero feedback from REX3’s non-food sectors to FABIO’s agricultural sectors. To reconcile differing regional classifications, we mapped FABIO’s ‘other uses’ of oil crops across 178 countries/regions to the detailed non-food sectors in REX3, covering most regions worldwide (Supplementary Table 2). Supply chain results and temporal changes relative to the 1995 baseline were calculated only for this subset of regions with consistent cross-database concordance (that is, ‘traceable supply chains’); regions that could not be mapped were omitted from the analysis.

### Supply chain impact mapping

To trace tele-connected biodiversity impacts from global oil crop cultivation through the entire supply chain, we applied the Supply Chain Impact Mapping (SCIM) methodology^18^ to oil crop cultivation and its downstream intermediate product and final-use sectors. SCIM is an MRIO-based approach that extends standard Leontief analysis by systematically tracking environmental impacts through distinct supply chain stages—from production, through intermediate processing, to final consumption. Whereas conventional consumption-based MRIO analysis directly links upstream production impacts to final demand, SCIM explicitly captures intermediate transformation steps. For instance, rather than directly linking biodiversity impacts from Brazilian soybean cultivation to Chinese meat consumption, SCIM tracks impacts through intermediate stages such as soybean cake processing in China. This framework has been applied to study greenhouse gas emissions in global plastics supply chains^30^ and biodiversity impacts in agri-food^39^ and mining supply chains^15^. The stage-wise mapping enables identification of strategic intervention points for targeted mitigation strategies.

In this study, we employed the SCIM method to construct two types of multidimensional array to examine tele-connected biodiversity impacts of food and non-food final uses separately. To analyse consumption patterns within food-use sectors, we developed a five-dimensional array using FABIO MRIO data from 1995 to 2020. This structure leverages the comprehensive agri-food supply chain detail inherent in the FABIO database. Each dimension captures a distinct perspective along the supply chain: the first details the ten specific oil crop cultivation sectors (production sectors); the second identifies the 192 countries or regions of cultivation (production regions); the third pinpoints the 192 countries or regions where consumption occurs (consumption regions); the fourth specifies the intermediate sectors involved in processing these oil crops (intermediate sectors) and the fifth represents the 123 FABIO sectors whose outputs are ultimately consumed (final-use sectors). The biodiversity impacts embodied in oil crop supply chains for food final uses shown in equation (2) is calculated as:

2 $${E}_{\mathrm{all}}^{\,\,\,\,\mathrm{food}}=\mathrm{diag}\left({D}_{\mathrm{pro}}\right)\times {L}_{\mathrm{pro}-T}\times \left({Y}_{T-\mathrm{all}}^{\,\,\,\,\,\,\,\,\,\,\mathrm{food}}+{A}_{T-O}\times {L}_{O-O}^{\,\,\,\,\,\,\,\,\,\,\,{\prime} }\times {Y}_{O-\mathrm{all}}^{\,\,\,\,\,\,\,\,\,\,\,\mathrm{food}}\right)$$

where *D*_pro_ represents the vector of global species loss coefficients (expressed in PSLglo per tonne of harvested oil crop), corresponding to each oil crop cultivation sector in each production region, calculated using the aforementioned detailed biodiversity impact assessment method. ${L}_{\mathrm{pro}-T}$ is the Leontief inverse referring to the cumulated inputs of the oil crop cultivation sectors into the oil crop economy (*T*, encompassing 6,144 sector–region combinations across oil crop cultivation, vegetable oil production and oil cake sectors in all regions). The expression in parentheses quantifies the total output required to meet global food demand, which consists of two components: (1) the direct final demand for oil crops (${Y}_{T-\mathrm{all}}^{\,\,\,\,\,\,\,\,\,\,\,\mathrm{food}}$) and (2) the intermediate demand for oil crops from other sectors (${A}_{T-O}\times {L}_{O-O}^{\,\,\,\,\,\,\,\,\,\,\,{\prime} }\times {Y}_{O-\mathrm{all}}^{\,\,\,\,\,\,\,\,\,\,\,\mathrm{food}}$). The intermediate demand component captures complex supply chain relationships, including: (1) the direct input from the oil crop economy into the non-oil crop economy (${A}_{T-O}$), (2) the cumulated inputs from the non-oil crop economy to itself (${L}_{O-O}^{\,\,\,\,\,\,\,\,\,\,\,{\prime} }$) and (3) the final demand for products from the non-oil crop economy (${Y}_{O-\mathrm{all}}^{\,\,\,\,\,\,\,\,\,\,\,\mathrm{food}}$). This framework accounts for various supply chain pathways, such as soybeans processed into soybean cake and subsequently used as livestock feed for meat production. The multidimensional arrays can be realized by diagonalizing *D*_pro_, ${Y}_{T-\mathrm{all}}^{\,\,\,\,\,\,\,\,\,\,\mathrm{food}}$ and ${Y}_{O-\mathrm{all}}^{\,\,\,\,\,\,\,\,\,\,\,\mathrm{food}}$ and by looping *Y*^food^ from different region combinations into equation (2).

To analyse biodiversity impacts of non-food final uses, we constructed five-dimensional arrays based on the hybrid MRIO model for 1995 to 2020. Unlike the arrays for food demand, the third dimension here represents 189 final consumption regions from REX3 and the fifth dimension contains 163 REX3 sectors instead of FABIO sectors. The biodiversity impacts embodied in oil crop supply chains of non-food final uses is calculated as:

3 $$\begin{array}{l}{E}_{\mathrm{all}}^{\,\,\,\,\mathrm{non}{\mbox{-}}\mathrm{food}}=\mathrm{diag}\left({D}_{\mathrm{pro}}\right)\times {L}_{\mathrm{pro}-T}\times \\ \left({A}_{\mathrm{other},T-\mathrm{rex}3}\times {L}_{{\rm{rex}}{3}}\times {Y}_{\mathrm{rex}3}+{A}_{T-O}\times {L}_{O-O}^{\,\,\,\,\,\,\,\,\,\,\,{\prime} }\times {A}_{\mathrm{other},O-\mathrm{rex}3}\times {L}_{\mathrm{rex}3}\times {Y}_{\mathrm{rex}3}\right)\end{array}$$

where *A*_other_ represents the coefficient matrix that links FABIO and REX3. *L*_rex3_ is the Leontief inverse matrix for REX3. *Y*_rex3_ denotes the final demand vector from the REX3 database. The multidimensional arrays we constructed can be realized by diagonalizing *D*_pro_ and *Y*_rex3_ and by loop *Y*_rex3_ from different region combinations into equation (3). All biodiversity impact calculations for food final uses and non-food final uses along oil crop supply chains are solved at country level and then aggregated to major regions for reporting. We further quantified the degree of biodiversity impact outsourcing via international trade as the share of consumption-based impacts occurring outside the consumption region (national scale). We computed outsourced shares separately for total, food and non-food final uses.

### Structural decomposition analysis

Structural decomposition analysis (SDA) is a widely recognized framework for quantifying the contributions of socio-economic decomposition factors to changes in environmental indicators. This study employs SDA to statistically attribute the change in biodiversity impacts arising from global oil crop cultivation in final consumption across diverse regions to four multiplicative factors. Specifically, we express the consumption-based biodiversity impacts *E* as:

4 $$E=\frac{E}{\mathrm{LU}}\times \frac{\mathrm{LU}}{Q}\times \frac{Q}{P}\times P=f\times {y}^{{\prime} }\times c\times P$$

where LU represents the required land-use area (total cultivated area of oil crops, hectare), *Q* denotes the total production of oil crops to meet final uses (tonne) and *P* is the population of the consumption region. From a consumption-based accounting perspective, the production quantity (*Q*) is equivalent to the total volume consumed. These variables are further used to define four drivers: *f* (the biodiversity impacts per hectare of land-use area, PSLglo per hectare) captures the average PSLglo of land-use area embodied in the final use of consuming regions, *y*′ (land-use area per tonne of production, hectare per tonne) reflects both yield changes within individual crops and shifts between different oil crops, *c* (per capita consumption, tonne) represents the average consumption volume per person of consuming regions and *P* (population), the total population of the consuming region, accounting for demographic scale effect. Data for *E*, LU and *Q* are derived from the SCIM analysis; *P* for each region is obtained from the World Population Prospects^55^. Accordingly, for each of the 2 years considered in the SDA (1995 and 2020), all four drivers are derived directly from the corresponding year-specific values of *E*, LU, *Q* and *P*.

The change in biodiversity impacts between two time periods *t*_0_ and *t*_1_ is given by equation (5):

5 $$\Delta E=E_{{t}_1}-E_{{t}_0}=f_{{t}_1}y\prime_{{t}_1}c_{{t}_1}P_{{t}_1}-f_{{t}_0}y\prime_{{t}_0}c_{{t}_0}P_{{t}_0}$$

The total change in biodiversity impacts can be decomposed into contributions from each factor as shown in equation (6):

6 $$\Delta E=\Delta {E}_{{{f}}}+\Delta {E}_{{{{y}}}^{{\prime} }}+\Delta {E}_{{{c}}}+\Delta {E}_{{{P}}}$$

We employed the complete decomposition approach^56^, also known as the Shapley/Sun decomposition^57,58^, which averages across all possible decomposition sequences (4! = 24). This method eliminates path dependency and symmetrically allocates interaction effects among factors without generating residuals^56,59^. For example, for the permutation *π* = (*f*, *y*′, *c*, *P*), the decomposition is:

7 $$\begin{array}{l}{{\varDelta} {E}}_{{\pi }}=\left({{f}}_{{t}_1}-{{f}}_{{t}_0}\right){{y}}_{{t}_0}^{\,\,\,{\prime} }{{c}}_{{t}_0}{{P}}_{{t}_0}+{{f}}_{{t}_1}\left({{y}}_{{t}_1}^{\,\,\,{\prime} }-{{y}}_{{t}_0}^{\,\,\,{\prime} }\right){{c}}_{{t}_0}{{P}}_{{t}_0}\\ \,\,\,\,\,\,\,\,\,\,\,\,\,+{{f}}_{{t}_1}{{y}}_{{t}_1}^{\,\,\,{\prime} }\left({{c}}_{{t}_1}-{{c}}_{{t}_0}\right){{P}}_{{t}_0}+{{f}}_{{t}_1}{{y}}_{{t}_1}^{\,\,\,{\prime} }{{c}}_{{t}_1}\left({{P}}_{{t}_1}-{{P}}_{{t}_0}\right)\end{array}$$

The contribution of each decomposition factor was then calculated as the average of its contributions across all permutations. We applied this SDA approach at both regional and global scales for the period 1995–2020, examining total consumption and food and non-food final uses. The analysis covered all oil crops combined, with separate assessments for oil palm and soybeans.

### Statistics and reproducibility

This study is a global modelling analysis based on a hybridized MRIO framework with spatially explicit land-use data; no experimental sampling, randomization or blinding is involved, and no data were excluded. Full input datasets and analysis code are publicly available or available upon reasonable request to ensure reproducibility.

### Reporting summary

Further information on research design is available in the Nature Portfolio Reporting Summary linked to this article.

## Supplementary information

Supplementary Information Supplementary Results 1–4, Discussion 1, Tables 1–8 and References.

Reporting Summary

## Source data

Source Data Fig. 1 Statistical source data workbook for Fig. 1, containing latitude-profile data, raster summary/crop totals, map-layer inventory and README notes.

Source Data Fig. 2 Statistical source data workbook for Fig. 2, containing Sankey linkage data, time series panel data, panel key and README notes.

Source Data Fig. 3 Statistical source data workbook for Fig. 3, containing food-use Sankey linkage data, stacked bar/marker panel data, axis/panel keys, stage map and README notes.

Source Data Fig. 4 Statistical source data workbook for Fig. 4, containing non-food-use Sankey linkage data, stacked bar/marker panel data, axis/panel keys, stage map and README notes.

Source Data Fig. 5 Statistical source data workbook for Fig. 5, containing structural decomposition analysis data for panels **a**–**i**, axis/panel keys and README notes.

## Data Availability

The primary datasets used in this study are publicly available from their respective sources. Spatial distribution data for oil crops were compiled using: SPAM 2020 (https://doi.org/10.7910/DVN/SWPENT), CROPGRIDS (https://doi.org/10.6084/m9.figshare.22491997) (ref. ^22^), remote-sensing-based global high-resolution spatial distribution data for oil palm (https://doi.org/10.5281/zenodo.11034130) (ref. ^24^) and closed-canopy coconut palm (https://doi.org/10.5281/zenodo.7453177) (ref. ^23^). Historical cropland area used for reconstructing annual oil crop maps (1995–2019) was derived from HYDE 3.5 (https://doi.org/10.24416/UU01-F45D44). Datasets used to calculate land-use intensity include the global crop-specific nitrogen fertilization dataset (https://doi.org/10.5281/zenodo.7408851) (ref. ^25^), the global area equipped for irrigation dataset (https://doi.org/10.5281/zenodo.6740334) (ref. ^27^) and the global crop-specific fertilization dataset for 1961–2019 (https://doi.org/10.6084/m9.figshare.25435432) (ref. ^26^). National-level historical harvested area and production data for each oil crop were sourced from the Food and Agriculture Organization Corporate Statistical Database (FAOSTAT, https://www.fao.org/faostat). Country and region boundary data were obtained from Natural Earth (https://www.naturalearthdata.com). The resolved REX3 multi-regional input–output (MRIO) data are available via Zenodo at https://doi.org/10.5281/zenodo.10354283 (ref. ^60^). The FABIO MRIO dataset can be accessed via Zenodo at https://doi.org/10.5281/zenodo.2577066 (ref. ^20^); the specific version FABIO v1.2 used in this study is available from the corresponding author upon reasonable request. Biodiversity impact factors used in the calculations are available via Zenodo at https://doi.org/10.5281/zenodo.10114492 (ref. ^29^). Population data were sourced from the United Nations World Population Prospects (https://population.un.org/wpp). All processed data and results generated during this study are available within the paper and its Supplementary Information, and the full dataset supporting the findings is deposited via Zenodo at https://doi.org/10.5281/zenodo.20081003 (ref. ^61^). Source data are provided with this paper.

## Code availability

All analysis code developed for this study, including scripts for harmonizing oil crop spatial distributions, computing spatially explicit biodiversity impact factors, performing the supply chain attribution via the hybridized MRIO framework and conducting the structural decomposition analysis is publicly available at https://github.com/ecological-systems-design/oilcrop-biodiversity-impact.
