# Supplementary material for: Oil crop supply chains drive rising global biodiversity loss and outsource impacts to the tropics
Source: Nat Food. 2026 Jun 19;7(7):655–68. doi: 10.1038/s43016-026-01375-4 (PMC13658035; doi:10.1038/s43016-026-01375-4)
Supplement: Supplementary file 1 [file 43016_2026_1375_MOESM1_ESM.pdf]

nature food

Supplementary information https://doi.org/10.1038/s43016-026-01375-4

Oil crop supply chains drive rising global biodiversity loss and outsource impacts to the tropics

In the format provided by the authors and unedited

SI Table of Contents

Results S1: Biodiversity impacts embodied in oil crop supply chains for food final uses

Results S2: Biodiversity impacts embodied in oil crop supply chains for non-food final uses

Results S3: Structural decomposition analysis (SDA) of biodiversity impact changes from oil crop cultivation (1995–2020)

Results S4: Robustness of temporal trends to land-system (crop distribution and LUI) assumptions

Discussion S1: Limitations and outlook of this study

Table S1: Types of 19 oil crops considered in this study

Table S2: Country/region concordance matrix for hybrid MRIO model

Table S3: Definition and aggregation of intermediate oil-crop sectors

Table S4: Sensitivity analysis of soybean induced biodiversity impacts (PSLglo) in 1995 under dynamic versus fixed-2020 assumptions

Table S5: Sensitivity analysis of oil palm-induced biodiversity impacts (PSLglo) in 1995 under dynamic versus fixed-2020 assumptions

Table S6: Sensitivity analysis of biodiversity impacts (PSLglo) from other aggregated oil crops in 1995 under dynamic versus fixed-2020 assumptions

Table S7: Global SDA driver contribution shares (Dynamic vs Fixed-2020)

Table S8: Monetary transaction matrix

SI References

Results S1: Biodiversity Impacts Embodied in Oil Crop Supply Chains for Food Final Uses

Figure 3 provides a comprehensive analysis of global oil crop supply chains, and their associated biodiversity impacts for food final uses from 1995 to 2020. Figure 3 is divided into two main components: a Sankey diagram (panels a–e) illustrating the interconnections of oil crop supply chains in 2020, and stacked bar charts (panels f–i) depicting temporal shifts in biodiversity impacts from various perspectives. In 2020, the biodiversity impact of oil crop cultivation for food final uses accounted for approximately 1.05% PSLglo, representing roughly 70% of the total. Among all oil crops, oil palm (~24%), soybeans (~24%) and coconuts (~23%), emerge as the dominant contributors to biodiversity loss. These primary crops are processed into various intermediate products. Vegetable oils account for approximately 51% of the biodiversity impacts for food final uses, with palm oil being the largest contributor (~21%). Soybeans are primarily processed into soybean cake (~14%), widely used as animal feed, while coconuts are predominantly consumed directly as fruit (~15%). Final consumption patterns reveal that many regions' consumption impacts are mainly caused by their region's production impacts, however China and the EU heavily depend on imports, highlighting a significant outsourcing of biodiversity impacts to Southeast Asia and Latin America as aforementioned (outlined in links between Figures 3b and 3c). A significant finding emerges regarding the indirect impact of animal-based food products on biodiversity loss. The widespread integration of oil crop byproducts, especially soybean cake, into animal feed establishes a clear link between livestock production and biodiversity degradation. Consequently, meat, dairy, and egg consumption collectively account for approximately 29% of the total biodiversity impacts for food final uses. This interconnection between plant-based and animal-based food systems underscores how dietary choices, particularly meat consumption patterns, significantly influence global biodiversity loss caused by oil crop cultivation.

From 1995 to 2020, the biodiversity impact driven by food final uses experienced significant growth, with oil palm cultivation being the primary driver, contributing approximately 42% to this increase. Soybean cultivation emerged as the second-largest contributor, accounting for about 31% of the growth, primarily in Brazil. As demonstrated in Figure 3f, biodiversity impacts across all oil crops have generally increased, mainly due to the surge in global demand for palm oil and soybean cake (Figures 3h–i). Notably, China's demand for soybean cake alone contributed approximately half of the total growth in demand for this product. In terms of food final uses, the intensification of global vegetable oil demand has amplified biodiversity impacts across all regions. Food consumption of vegetable oils was responsible for ~48% of the growth in biodiversity impacts, while meat consumption followed as the second largest contributor at ~27% (Figure 3i). China presents a particularly notable case, where biodiversity impact from meat consumption experienced a 7-fold increase from 1995 to 2020, more than half of the total growth in meat consumption-related impacts. Furthermore, while developed regions such as the EU continue to hold a substantial share of biodiversity impacts within the food system, over 90% of the growth in biodiversity impacts are driven by food consumption in developing regions. This trend indicates that developing regions, in their efforts to enhance nutritional profiles through increased consumption of vegetable oils and animal products derived from oil crops, are inadvertently amplifying the biodiversity impacts associated with oil crop cultivation. The findings underscore the complex interplay between improving

nutritional standards in developing countries and the global species loss consequences, highlighting the need for sustainable practices in oil crop production and consumption.

Results S2: Biodiversity Impacts Embodied in Oil Crop Supply Chains for Non-food Final Uses

Figure 4 provides a comprehensive analysis of global oil crop supply chains, and their associated biodiversity impacts for non-food final uses from 1995 to 2020. In 2020, oil crop cultivation for non-food final uses accounted for approximately 0.45% potential global species loss (PSLglo) in biodiversity impact, representing roughly 30% of the total impact. Among these crops, oil palm (~55%) and coconuts (~21%) emerges as the dominant contributors to biodiversity loss for non-food final uses. The geographical distribution of impacts is highly concentrated, with over 80% occurring in Asia and Latin America. Asia accounts for approximately 50% of the total biodiversity impact, with significant contributions from Indonesia (~22%), the Philippines (~13%), and Malaysia (~7%). Latin America contributes approximately 31%, primarily through Guatemala and Honduras (~7% each) and Brazil (~6%). Final consumption patterns reveal the EU as the largest market, responsible for about 21% of the total impact, followed by Latin America (~15%), the USA (~12%), and China (~12%). These intermediate products serve diverse industrial applications, with biochemicals (e.g., biodiesel) emerging as the most significant sector (~17% of impact), followed by the built environment (~13%), healthcare (~9%), and bioplastics (~7%). Notably, the EU dominates the bioplastics sector, accounting for approximately 63% of the associated biodiversity impacts.

Between 1995 and 2020, the biodiversity impacts caused by non-food final uses witnessed significant growth, almost doubling during this period. Approximately 93% of this increase was outsourced. Oil palm cultivation emerges as the primary driver, showing a 370% increase and contributing approximately 93% of the total impact growth (Figure 4f). This expansion primarily occurred in tropical regions, where oil palm is processed into palm oil and palm kernel oil for global industrial applications. From a consumption perspective, the EU demonstrated the most significant growth, contributing about 23% of the total increase in biodiversity impacts for non-food final uses (Figure 4h). China presents a particularly notable case, with its impact increasing approximately 5.4-fold, making it the second-largest contributor to impact growth after the EU (Figure 4h). Regional variations in use sectors reveal distinct patterns of industrial development. The EU's growth is primarily driven by the bioplastics industry, while China's expansion is concentrated in built environment and healthcare applications. In Latin America, Asia, and Africa, the biochemical sector demonstrates the most substantial growth. These trends underscore the expanding role of oil crop-derived products in non-food industries and highlight the diverse regional drivers behind this growth. The findings emphasize the critical need for sustainability initiatives in non-food sectors to mitigate global biodiversity loss associated with oil crop cultivation. Effective collaboration between production and consumption regions is essential to balance industrial development with environmental stewardship, ensuring that economic growth does not compromise global biodiversity conservation efforts. While the shift to oil-crop-based renewable materials and biofuels has clear climate benefits, it has intensified biodiversity loss by driving greater oil crop expansion in tropical regions, underscoring a critical trade-off in the pursuit of sustainable development.

Results S3: Structural Decomposition Analysis (SDA) of Biodiversity Impact Changes from Oil Crop Cultivation (1995–2020)

Figure 5 summarizes a structural decomposition analysis (SDA) of the change in consumption-based biodiversity impacts embodied in oil crop supply chains between 1995 and 2020. Following the decomposition in the main text, the change is partitioned into four multiplicative decomposition factors: biodiversity impacts per hectare of land use area, land use area per tonne of production, per-capita consumption, and population growth. SDA is an identity-based accounting decomposition; therefore, the results are interpreted as a statistical attribution of the observed change within the decomposition, rather than evidence of independent causal mechanisms. In Figure 5, panels (a–c) show total impacts for all oil crops, oil palm, and soybeans, respectively. Panels (d–f) repeat the decomposition for food final uses, and panels (g–i) for non-food final uses. Within each panel, stacked bars report the contribution of each decomposition factor (in % PSLglo) for the World and for individual regions. Negative contributions extend to the left of zero and indicate factors that statistically offset net impact growth. Hollow triangle markers indicate the net change (i.e., the sum of all factors). Percent labels shown in the World row report each factor's proportional contribution to the global net change; contributions can exceed 100% when positive and negative terms offset. For readability, the World row is displayed using a different x-axis scale than the regional rows.

Across the full oil crop supply chain, per-capita consumption is consistently the largest positive contributor to the global net change (World row). For total impacts (Figure 5a–c), per-capita consumption accounts for the dominant positive contribution, with population growth providing an additional positive contribution. These demand-side contributions are partly counterbalanced by a sizeable negative contribution from land use area per tonne of production, consistent with improved land-use efficiency (yield changes within crops and shifts in the oil crop mix). At the same time, biodiversity impacts per hectare of land use area contributes positively, indicating that the land use embodied in supply chains became associated with higher average per-hectare biodiversity impacts over time.

For food final uses (Figure 5d–f), the global pattern remains demand-dominated: per-capita consumption is the main positive contributor, while population growth also contributes positively. Negative contributions from land use area per tonne of production continue to provide substantial mitigation. The contribution from biodiversity impacts per hectare of land use area is small at the global level for food uses of all crops (2%) but is more pronounced for oil palm and soybeans (19 and 26%), consistent with changes in the per-hectare impact intensity of the land embodied in these food-oriented supply chains.

For non-food final uses (Figure 5g–i), demand-side contributions are even more prominent. Per-capita consumption contributes 109% of the global net change, reflecting strong growth in non-food demand. This increase is partly offset, especially for all crops combined, by a large negative contribution from land use area per tonne of production, again indicating improvements in land-use efficiency that mitigate (but do not eliminate) net impact growth. Biodiversity impacts per hectare of land use area contributes positively, suggesting that non-food supply chains increasingly became associated with higher per-hectare biodiversity

impacts. Population growth also adds a positive contribution, although smaller than per-capita consumption in most cases.

Beyond the global summary, Figure 5 shows distinct regional profiles. Regions with rapidly expanding demand (notably China and several other emerging economies) typically exhibit large positive contributions from per-capita consumption (and, in some cases, population growth), while the net change is partly offset by negative contributions from land use area per tonne of production. In Africa, population growth is consistently a major positive contributor, and the decomposition also indicates that land use area per tonne of production can contribute positively in some cases, consistent with weaker improvements in land-use efficiency relative to other regions. In many developed regions (e.g., the EU and the USA), the net changes tend to be smaller and reflect a balance between demand-side growth and efficiency-related mitigation; in the EU, the non-food panels highlight comparatively stronger demand-side contributions than the food panels. Across multiple panels, positive contributions from biodiversity impacts per hectare of land use area in import-oriented regions are consistent with increased association of their supply chains with land-use impacts occurring in higher-impact production areas.

Overall, the historical biodiversity impact growth along oil crop supply chains is statistically attributed primarily to demand expansion (per-capita consumption and population growth), while improvements captured by land use area per tonne of production provide substantial but incomplete mitigation. The additional positive contribution from biodiversity impacts per hectare of land use area highlights that where land-use pressures occur, and the per-hectare intensity of those pressures, remains a critical component of the observed change.

Results S4: Robustness of Temporal Trends to Land-system (Crop Distribution and LUI) Assumptions

To quantify how strongly our conclusions depend on the assumptions made for the crop spatial distribution and LUI time series, we additionally ran a counterfactual “Fixed-2020” scenario. In this counterfactual, both the crop map and the LUI classification (and the associated per-hectare species loss factors applied through the LUI classes) are held constant at their 2020 reference and back-cast to earlier years. This provides a bounding sensitivity test for the role of land-system dynamics (cropland expansion and changing LUI) in shaping our results. We find a clear two-level pattern: macro-level conclusions (global trends, commodity ranking, consumption-side patterns, and SDA attribution) are robust, whereas early-year country baselines can deviate substantially and heterogeneously across crops and countries.

Tables S4–S6 quantify how estimated 1995 country-level biodiversity impacts differ between our Dynamic (baseline) time series (year-specific cultivated-area maps combined with time-varying LUI) and the Fixed-2020 counterfactual that applies the 2020 crop map and LUI state to 1995. The results show that this modelling choice can substantially affect historical national accounting, with heterogeneous deviations across countries and crops. For soybeans (Table S4), Brazil’s 1995 impact is ~7% lower under the Dynamic time series than under Fixed-2020, whereas Argentina’s is ~34% higher. Additional large differences occur in Bolivia (~25% lower) and Uruguay (~47% higher). For oil palm (Table S5), deviations are relatively small for some key producers (e.g., Indonesia ~1%, Malaysia ~0%), but larger for others (e.g., Guatemala

~10% lower under Dynamic, Democratic Republic of the Congo ~8% higher, Mexico ~7% lower). For the aggregated “other oil crops” group (Table S6), differences remain visible in major contributors such as Indonesia (~3% higher), China (~7% higher), and Australia (~14% lower). Overall, these contrasts indicate that the Fixed-2020 approach can misrepresent 1995 baselines by implicitly applying the 2020 land-use profile to earlier years, whereas the Dynamic specification better reflects when and where land-use changes occurred over 1995–2020.

Despite the above baseline differences for specific countries and crops, the global temporal evolution of biodiversity impacts is virtually unchanged across scenarios. The global increase in biodiversity impacts (PSLglo) of oil-crop cultivation from 1995 to 2020 is +80% in the Dynamic time series versus +81% under the Fixed-2020 scenario. Crop-level attribution is likewise stable: oil palm accounts for about ~60% of the global increase in both scenarios, and soybeans for about ~25% in both. This indicates that the transgression of the biosphere integrity boundary identified in the main text is not an artifact of allowing LUI to vary over time, and that the dominance of oil palm and soybean is structurally evident under both assumptions.

Consumption-based patterns remain robust. Under both assumptions, emerging economies exhibit rapid growth in consumption footprints. For example, China’s consumption footprint increases 3.3-fold in the Dynamic time series versus 3.5-fold under Fixed-2020, while Africa’s increases 2.3-fold in both cases. This consistency reinforces our conclusion that the shifting centers of global biodiversity pressure are primarily dictated by changes in global trade flows and surging demand in emerging markets, which outweigh the effects of alternative LUI assumptions at the macro scale.

The robustness extends to the SDA conducted from a consumption-based perspective (Table S7). Globally, rising per-capita consumption remains the dominant driver of the net increase in biodiversity impacts between 1995 and 2020 (about 74% in the Dynamic time series versus about 73% under Fixed-2020), with population growth as the second largest driver (about 54% in both scenarios). Improvements in agricultural efficiency, captured by declining land use area per tonneof production, continue to offset roughly 43–44% of the net increase, while rising biodiversity impacts per hectare of land use contribute about 16–17%. For most regions (including major economies such as China, the EU, and Brazil), driver contribution shares differ by less than 5% across scenarios. Indonesia is an exception where relative contribution shares vary more strongly between scenarios because large positive and negative components largely cancel out and thus the net change is small. In the Dynamic time series, increasing per-capita consumption contributes +737% of Indonesia’s net change, largely offset by reductions in land use area per tonneof production (−615%) and biodiversity impacts per hectare of land use (−222%). Under Fixed-2020, the magnitudes shift (per-capita consumption +668%, offset by land use area per tonneof production −560% and biodiversity impacts per hectare of land use −190%), yet the substantive interpretation is unchanged: efficiency improvements and reductions in impacts per hectare play a critical role in dampening Indonesia’s consumption-based biodiversity impact despite strong growth in demand.

In sum, incorporating time-varying LUI is important for accurate historical country-level accounting (as highlighted by Tables S4–S6), but the aggregated and global temporal pattern, the relative importance of key commodities (notably oil palm and soybean), the consumption-side redistribution of biodiversity pressure, and the main SDA conclusions are robust to alternative LUI assumptions. All underlying data and full scenario results (Dynamic baseline and Fixed-2020 counterfactual) are publicly available to enable full transparency.

Discussion S1: Limitations and outlook of this study

Our study, while pioneering in its integration of spatially explicit biodiversity assessments with a hybridized MRIO framework and enhanced supply chain mapping, presents several limitations that point toward valuable avenues for future research.

First, although our approach substantially improves on previous global assessments by resolving within-country spatial heterogeneity and LUI, the reconstruction of historical oil crop distributions is still based on a spatially static and path-dependent assumption. We use the 2020 share of each oil crop within each 0.05° grid cell as an allocation weight and linearly scale this pattern backward and forward in time according to changes in total cropland area from the HYDE dataset. As a result, the spatial pattern of oil crop cultivation is effectively fixed and only its intensity changes over time, which limits our ability to represent migration of production areas, frontier expansion or abandonment, and crop replacement. Moreover, because historical values are computed as a 2020 baseline plus the HYDE differential multiplied by the 2020 weight, cells with zero oil crop area in 2020 (and thus zero weight) remain zero throughout the entire time series. This precludes us from reconstructing situations in which oil crops were cultivated earlier in the period but had disappeared by 2020, or where production shifted from one area to another. Finally, we allocate only the annual changes in total cropland from HYDE to oil crops based on their 2020 pattern, without explicitly using historical crop-specific harvested area statistics (e.g. from FAOSTAT), policy shifts, suitability changes or yield improvements. Future work should aim to combine time-series crop maps with crop-specific statistics and information on policy, biophysical suitability and technological change to better capture the dynamics of oil crop expansion, contraction and spatial reallocation.

Second, our analysis has focused solely on the biodiversity impacts from land occupation and has not accounted for land transformation. This limitation arises from the lack of temporal spatial data, which prevents us from identifying specific land-use changes (e.g., deforestation or conversion of pasture for soybean expansion). Integrating available time-series land use datasets, like HYDE[1] or LUH2[2], would enable a more comprehensive assessment that encompasses both land occupation and land transformation impacts on biodiversity, such as has been done for climate change impacts of bioeconomy[3]. In addition, while we used proportional allocation of land occupation impacts through the supply chain, alternative approaches, such as the marginal allocation method[4], should be explored in future analyses to provide insights into the impacts of marginal changes in production or consumption.

Third, our approach characterizes crop impacts using two land-use classes (cropland and plantation) combined with three LUI levels (minimal, light, and intense), following standard life cycle impact assessment (LCIA) practice and enabling spatially explicit assessment at the

ecoregion scale. While this framework does not capture all crop-intrinsic biodiversity responses within each land-use class, it does capture considerable heterogeneity across oil crops through ecoregion-resolved global species loss factors (PSLglo). These factors incorporate regional vulnerability and endemism, thereby distinguishing impacts based on the specific locations where each crop is cultivated. Recent studies have identified further crop-specific effects beyond spatial context, such as management practices and biological traits influencing species richness and community composition[5,6], indicating that certain aspects of variation remain unaccounted for in our analysis. At present, globally consistent, crop- and management-specific biodiversity response factors that also incorporate landscape context are lacking. Developing such comprehensive factors remains a critical research priority to enhance the precision of biodiversity footprinting in agricultural systems. In addition, the species loss factors we use are themselves static: they were derived by Scherer et al.[7] for a 2015 baseline and already incorporate the then prevailing patterns of habitat fragmentation and land-use intensity. We apply these factors unchanged over the 1995–2020 period, implicitly assuming that the relationships between land occupation, fragmentation and species loss remain constant within each ecoregion and LUI class. This simplification ignores potential temporal changes in landscape fragmentation, conservation measures and management practices, and thus may not fully capture the dynamics of extinction debt. Developing time-dependent biodiversity characterization factors that reflect evolving land-use patterns and habitat connectivity would be an important step towards more realistic dynamic biodiversity assessments.

Fourth, regarding our comparison with the planetary boundaries (PB)[8–10] framework, we acknowledge important methodological considerations. While we referenced recent work[11–13] proposing PSLglo-based thresholds for the biosphere integrity PB to contextualize our findings, directly adopting the same numerical threshold for both the annual rate of PSLglo (potential disappeared fraction of global species) and the original PB extinction rate metric (E/MSY, extinctions per million species per year) involves uncertainties due to fundamental conceptual differences. PSLglo captures extinction debt from ongoing habitat loss, whereas E/MSY measures realized extinction rates. Despite these complexities, our results clearly demonstrate that oil crop expansion exerts substantial pressures on global biosphere integrity. Future research should prioritize developing a robust empirical framework for a PSLglo-based PB to better integrate biodiversity indicators from life cycle assessment (LCA) into the PB framework, thereby providing clearer guidance for sustainable land use policy.

Fifth, in both Scherer et al. and our implementation, LUI is operationalized solely based on input intensity, combining information on irrigation extent and nitrogen and phosphorus fertilizer application, rather than on output-based measures such as yield or cropping frequency. This choice emphasizes the local pressure exerted on ecosystems per unit area, but it does not explicitly capture potential land-sparing effects of high-yield systems, where higher output per hectare could, in principle, reduce the total area required to meet a given level of demand. In our framework, such land-sparing effects are only represented indirectly through changes in harvested area over time, whereas the per-hectare biodiversity response is assumed to increase monotonically with input intensity. This conceptualization may lead to an overemphasis on high-input, high-yield systems in terms of per-hectare impacts, while

underrepresenting the biodiversity implications of extensive, low-input but land-demanding systems. Developing and testing alternative LUI metrics that integrate both input and output dimensions would therefore be a valuable avenue for future work.

Six, our biodiversity characterization operates at the level of terrestrial ecoregions. Within each ecoregion and land-use type/intensity class, all pixels are assigned the same per-hectare loss factor, which means that we cannot capture biodiversity gradients within ecoregions or explicitly single out very small frontier areas that may be disproportionately affected by recent expansion. Recent spatially explicit studies complement this perspective by quantifying multiple dimensions of local biodiversity at finer spatial resolution. For example, Li et al.[14] evaluate the impacts of future urban expansion on terrestrial vertebrates (amphibians, mammals and birds) at 10 × 10 km resolution using metrics such as within-site species richness and total abundance relative to an undisturbed baseline, as well as the numbers of threatened species and small-ranged species that may be lost in each grid cell. The spatially explicit assessment of cocoa cultivation impacts on biodiversity in Côte d’Ivoire further illustrates the value of fine-resolution indicators for identifying local hotspots associated with commodity expansion[15]. These indicators are highly relevant for conservation prioritization and hotspot mapping. However, such spatially explicit indicators differ from the LCIA endpoint metric used in our study (PSLglo), which is designed for consistent aggregation and comparison of biodiversity impacts across regions, crops and years and aligns with UNEP Life Cycle Initiative recommendations (and its use in global assessments such as the UNEP Global Resource Outlook[12]). Extending spatially explicit biodiversity indicators from case-study applications to harmonized global coverage (across multiple crops, regions and years), including consistent choices on baselines, taxonomic coverage and global normalization, would require substantial additional data and methodological development beyond the scope of this paper. Bridging these two strands of work, for instance by deriving globally normalized extinction-risk equivalents from spatially explicit state indicators, would allow future assessments to combine the strengths of fine-scale hotspot detection with the comparability and aggregability required in global biodiversity impact assessment.

Another limitation stems from the homogeneity assumptions inherent in integrating the REX3 and FABIO databases. Although our hybrid MRIO model represents a significant advancement by linking detailed supply chain data to biodiversity footprints, these simplifying assumptions might not fully capture the complexities of real-world supply chain dynamics. For instance, China's soybean sector features a dual system, comprising large-scale imports primarily intended as animal feed and a smaller, specialized domestic supply for human direct food consumption[16]. Such distinctions are not adequately reflected under the current integration approach. Future research should prioritize collecting and validating more granular supply chain data to better represent these relationships. Additionally, despite the relatively high resolution of our sectoral classification, biofuels are not treated as a separate sector. This limitation restricts our capacity to accurately track biodiversity impacts specifically associated with the biofuel industry. Enhancing sectoral resolution in future modeling efforts, particularly for emerging sectors like biofuels, will therefore be critical.

Table S1: Types of 19 oil crops considered in this study. This table presents a comprehensive overview of 19 oil crop types, which were consolidated into 10 Food and Agriculture Biomass Input-Output (FABIO)[17] sectors for supply chain analysis. The crops are categorized based on their growth habits into two distinct types: herbaceous and trees. This classification is essential for biodiversity impact assessment, as it determines the type of land use - tree crops correspond to plantation land use, while herbaceous crops correspond to cropland use. Among these crops, 14 are herbaceous plants (including castor beans, groundnuts, hempseed, linseed, melon seed, mustard seed, poppy seed, rapeseed, safflower seed, sesame seed, soybeans, sunflower seed, seed cotton, and other oilseeds), while 5 are tree crops (coconuts, karite nuts/shea nuts, oil palm fruit, olives, and tung nuts). The data sources column indicates the origin of global distribution data for each crop, utilizing three types of databases: remote sensing (RS), SPAM 2020[18], and CROPGRIDS[19]. Some crops, such as coconuts, employ multiple data sources - RS data for industrial plantations and SPAM 2020 for overall spatial distribution. This combination of data sources ensures comprehensive coverage and accurate representation of global oilseed crop distribution patterns. The FABIO sector classification demonstrates how these individual crops are grouped for supply chain analysis, with some crops maintaining their individual categories (like "Sesame seed" and "Sunflower seed"), while others are grouped under broader categories such as "Oilcrops, Other" or "Rape and Mustardseed."

Oil crop types	Growth Habit	FABIO Sector	FAOSTAT Sector	Data sources
Castor beans	herbaceous	Oilcrops, Other	Castor oil seeds	CROPGRIDS
Coconuts	tree	Coconuts - Incl Copra	Coconuts, in shell	SPAM 2020 + RS
Groundnuts in shell	herbaceous	Groundnuts (Shelled Eq)	Groundnuts, excluding shelled	SPAM 2020
Hempseed	herbaceous	Oilcrops, Other	Hempseed	CROPGRIDS
Karite nuts (shea nuts)	tree	Oilcrops, Other	Karite nuts (sheanuts)	CROPGRIDS
Linseed	herbaceous	Oilcrops, Other	Linseed	CROPGRIDS
Melon seed	herbaceous	Oilcrops, Other	Melonseed	CROPGRIDS
Mustard seed	herbaceous	Rape and Mustardseed	Mustard seed	CROPGRIDS
Oil palm fruit	tree	Oil, palm fruit	Oil palm fruit	RS
Oilseeds, other	herbaceous	Oilcrops, Other	Other oil seeds, n.e.c.	CROPGRIDS
Olives	tree	Olives (including preserved)	Olives	CROPGRIDS
Poppy seed	herbaceous	Oilcrops, Other	Poppy seed	CROPGRIDS
Rapeseed	herbaceous	Rape and Mustardseed	Rape or colza seed	SPAM 2020
Safflower seed	herbaceous	Oilcrops, Other	Safflower seed	CROPGRIDS
Sesame seed	herbaceous	Sesame seed	Sesame seed	SPAM 2020
Soybeans	herbaceous	Soyabeans	Soyabeans	SPAM 2020
Sunflower seed	herbaceous	Sunflower seed	Sunflower seed	SPAM 2020
Tung nuts	tree	Oilcrops, Other	Tung nuts	CROPGRIDS
Seed Cotton	herbaceous	Seed cotton	Seed Cotton	SPAM 2020

Table S2: Country/region concordance matrix for hybrid MRIO model. The table includes the following columns: Country/region names in FABIO: The country/region names as represented in the FABIO database; ISO3 code: The three-letter country/region code corresponding to each country; Country/region names in REX3: The country/region names as represented in the REX3 database. If a country/region is marked as NA in this column, it indicates that no direct match exists between FABIO and REX3 for that region. This concordance matrix was utilized to allocate FABIO's final demand category "other uses" across REX3 manufacturing sectors based on sectoral use shares derived from REX3. The final hybridized MRIO framework includes 178 regions, covering 98% of the global biodiversity impact of global oil crop cultivation.

Country/region names in FABIO	ISO3 code	Country/region names in REX3
Armenia	ARM	Armenia
Afghanistan	AFG	Afghanistan
Albania	ALB	Albania
Algeria	DZA	Algeria
Angola	AGO	Angola
Antigua and Barbuda	ATG	Antigua
Argentina	ARG	Argentina
Australia	AUS	Australia
Austria	AUT	Austria
Bahamas	BHS	Bahamas
Bahrain	BHR	Bahrain
Barbados	BRB	Barbados
Belgium-Luxembourg	BLX	NA
Bangladesh	BGD	Bangladesh
Bolivia (Plurinational State of)	BOL	Bolivia
Botswana	BWA	Botswana
Brazil	BRA	Brazil
Belize	BLZ	Belize
Solomon Islands	SLB	NA
Brunei Darussalam	BRN	Brunei
Bulgaria	BGR	Bulgaria
Myanmar	MMR	Myanmar
Burundi	BDI	Burundi
Cameroon	CMR	Cameroon
Canada	CAN	Canada
Cabo Verde	CPV	Cape Verde
Central African Republic	CAF	Central African Republic
Sri Lanka	LKA	Sri Lanka
Chad	TCD	Chad
Chile	CHL	Chile
China, mainland	CHN	China
Colombia	COL	Colombia
Congo	COG	Congo
Costa Rica	CRI	Costa Rica
Cuba	CUB	Cuba
Cyprus	CYP	Cyprus
Czechoslovakia	CSK	NA
Azerbaijan	AZE	Azerbaijan
Benin	BEN	Benin
Denmark	DNK	Denmark
Dominica	DMA	Dominican Republic

Dominican Republic	DOM	Dominican Republic
Belarus	BLR	Belarus
Ecuador	ECU	Ecuador
Egypt	EGY	Egypt
El Salvador	SLV	El Salvador
Estonia	EST	Estonia
Fiji	FJI	Fiji
Finland	FIN	Finland
France	FRA	France
French Polynesia	PYF	French Polynesia
Djibouti	DJI	Djibouti
Georgia	GEO	Georgia
Gabon	GAB	Gabon
Gambia	GMB	Gambia
Germany	DEU	Germany
Bosnia and Herzegovina	BIH	Bosnia and Herzegovina
Ghana	GHA	Ghana
Kiribati	KIR	NA
Greece	GRC	Greece
Grenada	GRD	NA
Guatemala	GTM	Guatemala
Guinea	GIN	Guinea
Guyana	GUY	Guyana
Haiti	HTI	Haiti
Honduras	HND	Honduras
China, Hong Kong SAR	HKG	Hong Kong
Hungary	HUN	Hungary
Croatia	HRV	Croatia
Iceland	ISL	Iceland
India	IND	India
Indonesia	IDN	Indonesia
Iran (Islamic Republic of)	IRN	Iran
Iraq	IRQ	Iraq
Ireland	IRL	Ireland
Israel	ISR	Israel
Italy	ITA	Italy
Côte d'Ivoire	CIV	Côte d'Ivoire
Kazakhstan	KAZ	Kazakhstan
Jamaica	JAM	Jamaica
Japan	JPN	Japan
Jordan	JOR	Jordan
Kyrgyzstan	KGZ	Kyrgyzstan

Kenya	KEN	Kenya
Cambodia	KHM	Cambodia
Democratic People's Republic of Korea	PRK	North Korea
Republic of Korea	KOR	South Korea
Kuwait	KWT	Kuwait
Latvia	LVA	Latvia
Lao People's Democratic Republic	LAO	Laos
Lebanon	LBN	Lebanon
Lesotho	LSO	Lesotho
Liberia	LBR	Liberia
Libya	LBY	Libya
Lithuania	LTU	Lithuania
China, Macao SAR	MAC	Macao SAR
Madagascar	MDG	Madagascar
Malawi	MWI	Malawi
Malaysia	MYS	Malaysia
Maldives	MDV	Maldives
Mali	MLI	Mali
Malta	MLT	Malta
Mauritania	MRT	Mauritania
Mauritius	MUS	Mauritius
Mexico	MEX	Mexico
Mongolia	MNG	Mongolia
Morocco	MAR	Morocco
Mozambique	MOZ	Mozambique
Republic of Moldova	MDA	Moldova
Namibia	NAM	Namibia
Nepal	NPL	Nepal
Netherlands	NLD	Netherlands
Netherlands Antilles	ANT	Netherlands Antilles
New Caledonia	NCL	New Caledonia
The former Yugoslav Republic of Macedonia	MKD	TFYR Macedonia
Vanuatu	VUT	Vanuatu
New Zealand	NZL	New Zealand
Nicaragua	NIC	Nicaragua
Niger	NER	Niger
Nigeria	NGA	Nigeria
Norway	NOR	Norway
Pakistan	PAK	Pakistan
Panama	PAN	Panama
Czech Republic	CZE	Czech Republic
Papua New Guinea	PNG	Papua New Guinea

Paraguay	PRY	Paraguay
Peru	PER	Peru
Philippines	PHL	Philippines
Poland	POL	Poland
Portugal	PRT	Portugal
Guinea-Bissau	GNB	NA
Timor-Leste	TLS	NA
Puerto Rico	PRI	NA
Eritrea	ERI	Eritrea
Qatar	QAT	Qatar
Zimbabwe	ZWE	Zimbabwe
Romania	ROU	Romania
Rwanda	RWA	Rwanda
Russian Federation	RUS	Russia
Serbia and Montenegro	SCG	NA
Saint Kitts and Nevis	KNA	NA
Saint Lucia	LCA	NA
Saint Vincent and the Grenadines	VCT	NA
Sao Tome and Principe	STP	Sao Tome and Principe
Saudi Arabia	SAU	Saudi Arabia
Senegal	SEN	Senegal
Sierra Leone	SLE	Sierra Leone
Slovenia	SVN	Slovenia
Slovakia	SVK	Slovakia
Singapore	SGP	Singapore
Somalia	SOM	Somalia
South Africa	ZAF	South Africa
Spain	ESP	Spain
Suriname	SUR	Suriname
Tajikistan	TJK	Tajikistan
Swaziland	SWZ	Swaziland
Sweden	SWE	Sweden
Switzerland	CHE	Switzerland
Syrian Arab Republic	SYR	Syria
Turkmenistan	TKM	Turkmenistan
China, Taiwan Province of	TWN	Taiwan
United Republic of Tanzania	TZA	Tanzania
Thailand	THA	Thailand
Togo	TGO	Togo
Trinidad and Tobago	TTO	Trinidad and Tobago
Oman	OMN	Oman
Tunisia	TUN	Tunisia

Turkey	TUR	Turkey
United Arab Emirates	ARE	UAE
Uganda	UGA	Uganda
USSR	SUN	Former USSR
United Kingdom	GBR	UK
Ukraine	UKR	Ukraine
United States of America	USA	USA
Burkina Faso	BFA	Burkina Faso
Uruguay	URY	Uruguay
Uzbekistan	UZB	Uzbekistan
Venezuela (Bolivarian Republic of)	VEN	Venezuela
Viet Nam	VNM	Viet Nam
Ethiopia	ETH	Ethiopia
Samoa	WSM	Samoa
Yugoslav SFR	YUG	NA
Yemen	YEM	Yemen
Democratic Republic of the Congo	COD	DR Congo
Zambia	ZMB	Zambia
Belgium	BEL	Belgium
Luxembourg	LUX	Luxembourg
Serbia	SRB	Serbia
Montenegro	MNE	Montenegro
Sudan	SDN	Sudan
South Sudan	SSD	South Sudan
RoW	ROW	RoW

Table S3: Definition and aggregation of intermediate oil-crop sectors. Table S3 lists the intermediate FABIO sectors used to represent the oil-crop economy, groups them into three processing classes (Direct forms, Vegetable oils, and Cakes), and documents how each sector is aggregated to the intermediate processing labels shown in the Overall/Food and Non-food Sankey diagrams (e.g., “Other Direct Form”, “Other Vegetable Oils”, and “Other Cake”). This table is provided to clarify the constituents of the “Other …” categories in Figures 2–4 and to ensure transparency and reproducibility of the aggregation.

Sector code	Sector	Class	Overall/Food (Figures 2-3 label)	Non-food (Figure 4 label)
**c021**	Soyabeans	Direct forms	Other Direct Form	Other Direct Form
**c022**	Groundnuts	Direct forms	Other Direct Form	Other Direct Form
**c023**	Sunflower seed	Direct forms	Other Direct Form	Other Direct Form
**c024**	Rape and Mustardseed	Direct forms	Other Direct Form	Other Direct Form
**c025**	Seed cotton	Direct forms	Other Direct Form	Seed Cotton
**c026**	Coconuts - Incl Copra	Direct forms	Coconut Fruit	Coconut Fruit
**c027**	Sesame seed	Direct forms	Other Direct Form	Other Direct Form

**c028**	Oil, palm fruit	Direct forms	Other Direct Form	Other Direct Form
**c029**	Olives (including preserved)	Direct forms	Olive Fruit	Other Direct Form
**c030**	Oilcrops, Other	Direct forms	Other Direct Form	Other Direct Form
**c068**	Soyabean Oil	Vegetable oils	Soybean Oil	Soybean Oil
**c069**	Groundnut Oil	Vegetable oils	Other Vegetable Oils	Other Vegetable Oils
**c070**	Sunflowerseed Oil	Vegetable oils	Other Vegetable Oils	Other Vegetable Oils
**c071**	Rape and Mustard Oil	Vegetable oils	Other Vegetable Oils	Other Vegetable Oils
**c072**	Cottonseed Oil	Vegetable oils	Other Vegetable Oils	Other Vegetable Oils
**c073**	Palmkernel Oil	Vegetable oils	Other Vegetable Oils	Palm Kernel Oil
**c074**	Palm Oil	Vegetable oils	Palm Oil	Palm Oil
**c075**	Coconut Oil	Vegetable oils	Coconut Oil	Coconut Oil
**c076**	Sesameseed Oil	Vegetable oils	Other Vegetable Oils	Other Vegetable Oils
**c077**	Olive Oil	Vegetable oils	Olive Oil	Other Vegetable Oils
**c080**	Oilcrops Oil, Other	Vegetable oils	Other Vegetable Oils	Other Vegetable Oils
**c081**	Soyabean Cake	Cakes	Soybean Cake	Soybean Cake
**c082**	Groundnut Cake	Cakes	Other Cake	Other Cake
**c083**	Sunflowerseed Cake	Cakes	Other Cake	Other Cake
**c084**	Rape and Mustard Cake	Cakes	Other Cake	Other Cake
**c085**	Cottonseed Cake	Cakes	Other Cake	Other Cake
**c086**	Palmkernel Cake	Cakes	Other Cake	Other Cake
**c087**	Copra Cake	Cakes	Other Cake	Other Cake
**c088**	Sesameseed Cake	Cakes	Other Cake	Other Cake
**c089**	Oilseed Cakes, Other	Cakes	Other Cake	Other Cake

Table S4: Sensitivity analysis of soybean induced biodiversity impacts (PSLglo) in 1995 under dynamic versus constant assumptions. The table presents the top 10 countries (ranked by their 2020 impact) and the Rest of the World, comparing impacts calculated using actual 1995 parameters (Dynamic) against a scenario applying 2020 parameters to 1995 (Fixed-2020). "% Difference" indicates the deviation of the dynamic baseline from the constant assumption. Values represent biodiversity impact (unit: 10^{-8} % PSLglo).

Country	2020	1995 (Dynamic)	1995 (Fixed-2020)	Growth (vs Dynamic)	Growth (vs Fixed-2020)	1995 Scenario Diff
**Brazil**	16523541	4834126	5186984	242%	219%	-7%
**Argentina**	2066895	979869	733974	111%	182%	34%
**United States of America**	2007984	1576361	1496064	27%	34%	5%
**India**	1541313	637208	636488	142%	142%	0%
**China, mainland**	1523397	1320126	1252739	15%	22%	5%
**Paraguay**	1342791	271416	271999	395%	394%	-0%
**Nigeria**	325258	159985	166165	103%	96%	-4%
**Bolivia**	262924	62222	82929	323%	217%	-25%
**South Africa**	256688	22144	23666	1059%	985%	-6%
**Uruguay**	187566	2564	1739	7215%	10686%	47%

**Rest of the World**	1657765	2325448	2335378	-29%	-29%	-0%

Table S5: Sensitivity analysis of oil palm-induced biodiversity impacts (PSLglo) in 1995 under dynamic versus constant assumptions. The table presents the top 10 countries (ranked by their 2020 impact) and the Rest of the World, comparing impacts calculated using actual 1995 parameters (Dynamic) against a scenario applying 2020 parameters to 1995 (Fixed-2020). The units are consistent with Table S4.

Country	2020	1995 (Dynamic)	1995 (Fixed-2020)	Growth (vs Dynamic)	Growth (vs Fixed-2020)	1995 Scenario Diff
**Indonesia**	12915710	1067594	1053686	1110%	1126%	1%
**Malaysia**	7596793	3678986	3688353	106%	106%	-0%
**Guatemala**	5181002	282679	314000	1733%	1550%	-10%
**Honduras**	4723708	683542	697309	591%	577%	-2%
**Ecuador**	2797286	1298984	1350784	115%	107%	-4%
**Democratic Republic of the Congo**	2109552	1660666	1544490	27%	37%	8%
**Cameroon**	1951016	424708	444053	359%	339%	-4%
**Nigeria**	1915763	1416176	1448107	35%	32%	-2%
**Papua New Guinea**	1866391	526855	524495	254%	256%	0%
**Mexico**	1547138	38378	41405	3931%	3637%	-7%
**Rest of the World**	8538465	2414655	2382704	254%	258%	1%

Table S6: Sensitivity analysis of biodiversity impacts (PSLglo) from other aggregated oil crops in 1995 under dynamic versus constant assumptions. The table presents the top 10 countries (ranked by their 2020 impact) and the Rest of the World, comparing impacts calculated using actual 1995 parameters (Dynamic) against a scenario applying 2020 parameters to 1995 (Fixed-2020). The units are consistent with Table S4.

Country	2020	1995 (Dynamic)	1995 (Fixed-2020)	Growth (vs Dynamic)	Growth (vs Fixed-2020)	1995 Scenario Diff
**Indonesia**	13850527	13634319	13252264	2%	5%	3%
**Philippines**	10494791	8967532	8984321	17%	17%	-0%
**India**	6132397	6079730	6170020	1%	-1%	-1%
**China, mainland**	4223615	4859852	4546308	-13%	-7%	7%
**Spain**	3715009	3228623	3235453	15%	15%	-0%
**United Republic of Tanzania**	2951588	1742790	1752092	69%	68%	-1%
**Australia**	2438983	748033	865582	226%	182%	-14%
**Papua New Guinea**	1878861	2575037	2562703	-27%	-27%	0%
**Nigeria**	1541851	764145	746161	102%	107%	2%
**Myanmar**	1471154	873353	884983	68%	66%	-1%
**Rest of the World**	23978900	20975310	21055385	14%	14%	-0%

Table S7: Global SDA driver contribution shares (Dynamic vs Fixed-2020). This table reports, for each consumption region (including World) in the consumption-based SDA (Figure 5a; Total, all oil crops), the percentage contribution of the four drivers f (biodiversity impacts per hectare of land use area), y′ (land use area per tonne of production), c (per-capita consumption), and P (population) to the net change in consumption-based biodiversity impacts between 1995 and 2020. Results are shown for the Dynamic baseline (year-specific crop spatial distributions and LUI) and the counterfactual Fixed-2020 scenario (holding crop spatial distributions and LUI constant at their 2020 reference); Δ denotes the difference in contribution shares (Fixed-2020 minus Dynamic) in percentage points. All values are rounded to integer percentages; shares can be negative or exceed ±100% when positive and negative contributions offset.

Region	Dynamic f	Dynamic y′	Dynamic c	Dynamic P	Fixed-2020 f	Fixed-2020 y′	Fixed-2020 c	Fixed-2020 P	Δ f (%)	Δ y′ (%)	Δ c (%)	Δ P (%)
World	16	-44	74	55	17	-43	73	53	1	0	0	0
Africa	-2	71	-45	77	-3	72	-46	77	-1	0	0	0
Indonesia	-222	-615	737	200	-190	-560	668	182	31	55	-68	-18
India	13	-114	135	67	11	-116	138	68	-2	-2	3	1
Philippines	645	865	314	-1724	581	763	277	-1520	-64	-102	-37	204
China	19	-52	119	15	22	-51	114	15	3	2	-5	-1
Other Asia	16	-27	53	58	17	-26	52	57	1	0	-1	-1
EU	78	-175	175	21	79	-174	174	21	0	1	-1	0
Other Europe	30	-77	127	19	30	-76	127	19	0	0	-1	0
Brazil	25	-111	140	46	22	-114	145	48	-3	-4	5	2
Other Latin America	19	26	16	38	18	27	16	39	-1	0	0	1
USA	116	-171	74	81	116	-167	72	79	0	4	-2	-2
Other North America	-1495	1741	355	-501	-1279	1505	307	-433	216	-236	-48	67
Oceania	52	101	-98	46	50	104	-101	47	-2	3	-3	1

Table S8: Monetary transaction matrix. Table S8 provides the supply and use filter matrices used to compute the monetary transaction matrix. These matrices were applied to allocate financial flows accurately within the hybridized MRIO framework. This table is provided as a standalone Excel file to ensure clarity and ease of reference. The supply and use filters define sectoral relationships, facilitating the integration of monetary transactions into the overall modeling framework.

References

1. Klein Goldewijk, K., Beusen, A., Doelman, J. & Stehfest, E. Anthropogenic land use estimates for the Holocene – HYDE 3.2. *Earth Syst. Sci. Data* **9**, 927–953 (2017).
2. Hurtt, G. C. *et al.* Harmonization of global land use change and management for the period 850–2100 (LUH2) for CMIP6. *Geosci. Model Dev.* **13**, 5425–5464 (2020).
3. Cabernard, L., Schwingshackl, C., Pfister, S. & Hellweg, S. Rising greenhouse gas emissions embodied in the global bioeconomy supply chain. *Commun. Earth Environ.* **6**, 1–14 (2025).
4. Cabernard, L., Pfister, S. & Hellweg, S. Biodiversity impacts of recent land-use change driven by increases in agri-food imports. *Nat. Sustain.* **7**, 1512–1524 (2024).
5. Núñez-Regueiro, M. M., Siddiqui, S. F. & Fletcher Jr, R. J. Effects of bioenergy on biodiversity arising from land-use change and crop type. *Conserv. Biol.* **35**, 77–87 (2021).
6. Fan, S. *et al.* Impact of Crop Type on Biodiversity Globally. *Glob. Change Biol.* **30**, e70005 (2024).
7. Scherer, L. *et al.* Biodiversity Impact Assessment Considering Land Use Intensities and Fragmentation. *Environ. Sci. Technol.* **57**, 19612–19623 (2023).
8. Rockström, J. *et al.* Planetary Boundaries: Exploring the Safe Operating Space for Humanity. *Ecol. Soc.* **14**, (2009).
9. Steffen, W. *et al.* Planetary boundaries: Guiding human development on a changing planet. *Science* **347**, 1259855 (2015).
10. Richardson, K. *et al.* Earth beyond six of nine planetary boundaries. *Sci. Adv.* **9**, eadh2458 (2023).
11. Nathani, C. *et al.* Environmental hotspots in the supply chain of Swiss companies. *Rütter Soceco AG Treeze Ltd Uster Switz.* (2019).

12. Programme, U. N. E. & Panel, I. R. *Global Resources Outlook 2024: Bend the Trend – Pathways to a Liveable Planet as Resource Use Spikes*. https://wedocs.unep.org/20.500.11822/44901 (2024).

13. Cabernard, L., Pfister, S. & Hellweg, S. Biodiversity impacts of recent land-use change driven by increases in agri-food imports. *Nat. Sustain.* **7**, 1512–1524 (2024).

14. Li, G. *et al.* Global impacts of future urban expansion on terrestrial vertebrate diversity. *Nat. Commun.* **13**, 1628 (2022).

15. Wang, S. & Pfister, S. Landscape-Scale Biodiversity Impacts Analysis of Côte d'Ivoire's Cocoa Cultivation along Export Supply Chains. *Environ. Sci. Technol.* **58**, 9601–9611 (2024).

16. Wang, M., Liu, D., Wang, Z. & Li, Y. Structural Evolution of Global Soybean Trade Network and the Implications to China. *Foods* **12**, 1550 (2023).

17. Bruckner, M. *et al.* FABIO—The Construction of the Food and Agriculture Biomass Input–Output Model. *Environ. Sci. Technol.* **53**, 11302–11312 (2019).

18. Institute (IFPRI), I. F. P. R. Global Spatially-Disaggregated Crop Production Statistics Data for 2020 Version 1.0. Harvard Dataverse https://doi.org/10.7910/DVN/SWPENT (2024).

19. Tang, F. H. M. *et al.* CROPGRIDS: a global geo-referenced dataset of 173 crops. *Sci. Data* **11**, 413 (2024).
